# Supplementary material for: Ectopic Expression of Distinct PLC Genes Identifies ‘Compactness’ as a Possible Architectural Shoot Strategy to Cope with Drought Stress
Source: Plant Cell Physiol. 2023 Oct 16;65(6):885–903. doi: 10.1093/pcp/pcad123 (PMC11209554; doi:10.1093/pcp/pcad123)
Supplement: pcad123_Supp [file pcad123_supp.zip › suppl_data/pcp-2023-e-00130-File012.pdf]

Overview supplemental data

van Hooren et al. 2021 JXB

Supplemental Table S1. Primers for the construction of PLC9 OE and tor the PLC9 qPCR
Supplemental Table S2. Promoters used for Tissue Specific Expression of *PLC5*
Supplemental Table S3. Key amino acids of the catalytic site of rat PLCδ1 and their counterparts in Arabidopsis PLCs
Supplemental Figure 1. PCA plot of Chlorophyl fluorescence camera traits.
Supplemental Figure 2. Darkyield of plants at control- and water limiting conditions.
Supplemental Figure 3. Eccentricity of plants at control- and water limiting conditions.
Supplemental Figure 4. F_0 of plants at control- and water limiting conditions.
Supplemental Figure 5. F_m of plants at control- and water limiting conditions
Supplemental Figure 6. F_v/F_m ratios of plants at control- and water limiting conditions.
Supplemental Figure 7. Insidediameter of plants at control- and water limiting
Supplemental Figure 8. Midyieldfluoresence of plants at control- and water limiting conditions.
Supplemental Figure 9. Minimal leaf diameter of plants at control- and water limiting conditions.
Supplemental Figure 10. Outside leaf diameter of plants at control- and water limiting conditions.
Supplemental Figure 11. Average leaf diameter of plants at control- and water limiting conditions.
Supplemental Figure 12. Number of leaves of plants at control- and water limiting conditions.
Supplemental figure 13. Plant perimeter at control- and water limiting conditions.
Supplemental figure 14. Area by circumference at control- and water limiting conditions
Supplemental figure 15. Stockiness at control- and water limiting conditions.
Supplemental figure 16. Hue at control- and water limiting conditions.
Supplemental figure 17. Brightness at control- and water limiting conditions.
Supplemental figure 18. Saturation at control- and water limiting conditions
Supplemental figure 19. Fresh- and dry weight and relative water content in WT and PLC-OE lines.
Supplemental figure 20. Projected plant surface area of TSEP lines under control and water limiting conditions.
Supplemental figure 21. Fresh- and dry weight and relative water content in WT and TSEP lines.
Supplemental figure 22. Convex hull permiter at control- and water limiting conditions
Supplemental figure 23. Convex hull area at control- and water limiting conditions.
Supplemental figure 24. Compactness at control- and water limiting conditions.
Supplemental figure 25. Surface coverage at control- and water limiting conditions.
Supplemental figure 26. RGB Blue values at control- and water limiting conditions
Supplemental figure 27. RGB Green values at control- and water limiting conditions
Supplemental figure 28. RGB Red values at control- and water limiting conditions

Supplemental Table S1. Primers for the construction of PLC9 OE and tor the *PLC9* qPCR

Primers	Sequence
PLC9_fwd	ggggacaagtttgtacaaaaaagcaggctATGGTGAATTTAAGAAAGAAGTTTG
PLC9_rev	ggggaccactttgtacaagaaagctgggtTCTAAGACCACTTAAAACGTGTGAGC
attB1-PLC2fw	ggggacaagtttgtacaaaaaagcaggctATGTCGAAGCAAACGTAC
attB2-PLC2rv	ggggaccactttgtacaagaaagctgggtCACAAACTCCACCTTCACGA
LB1	GCCTTTTCAGAAATGGATAAATAGCCTTGCTTCC
PLC9_qPCR_fwd	ATCGTGACATGAATGCTCCA
PLC9_qPCR_rev	ACGCATATGCCGTCTTTACC
Q_PLC2_set1_fw	TTTGGAGGATTTGTATGTGTGAGAG
Q_PLC2_set1_rv	CCACAAATCATCATACGATTCAAGTG
SAND_qPCR_fwd	CAGACAAGGCGATGGCGATA
SAND_qPCR_rev	GCTTTCTCTCAAGGGTTTCTGGGT

Supplemental Table S2. Promoters used for Tissue Specific Expression of *PLC5*

Promotor	Expression	Corresponding gene	
A14	LRC, atrichoblast, trichromes	AT5G43040	KCS6
Cab3	Mesophyll	AT1G29910	LHCB1.2
CER6	Guard cells (epidermis leaves)	AT1G68530	Cysteine/Histidine-rich C1 domain family protein
E28	Endodermis, pericycle	AT4G00940	DOF4.1
E30	Endodermis in mature root	AT4G21340	B70
E49	Cortex in elongation zone	AT3G05150	Major facilitator superfamily protein
LRC1	Lateral root cap, atrichoblast in root, epidermis in shoot	AT1G08930	ERD6
LRC3	Lateral root cap, columella	AT1G30650	WRKY14
ML1-1	epidermis in root and shoot	AT4G21750	ATML1
Q4	Quiescent centre	AT1G69490	ANAC029
S1	Epidermis root, pericycle in late EZ	AT4G27410	RD26
S2	Stele	AT3G61850	DAG1
DR5	Auxin maxima		

Supplemental Table S3. Key amino acids of the catalytic site of rat PLCδ1 and their counterparts in Arabidopsis PLCs. Differences are given in red. Based on Ellis et al. (1998)

	Catalytic site								
RnPLCδ1	H:311	H:356	Y:155	E:341	D:343	E:390	K:440	S:522	R:549
AtPLC1	H:120	H:166	F:352	E:150	D:152	E:200	K:249	S	R
AtPLC2	H121	H:164	Y:375	E:148	D:150	E:198	K:248	S	R
AtPLC3	H:118	H:167	Y:354	E:151	D:153	E:201	K:250	S	R
AtPLC4	H:129	H:174	Y:391	E:159	D:161	E:208	K:257	S	R
AtPLC5	H:127	H:173	Y:372	E:157	D:159	E:207	K:256	S	R
AtPLC6	H:152	H:198	Y:407	E:182	D:184	E:232	K:281	S	R
AtPLC7	H:118	H:164	Y:381	E:148	D:150	E:198	K:248	S	R
AtPLC8	L:120	P:164	R:327	E:149	D:151	K:198	R:247	-	R
AtPLC9	L:122	P:168	R:326	E:153	D:155	K:203	R:253	-	R

Supplemental table 4 - Significance of traits in phenomics experiment

All statistics was done with anova, followed by a TUKEY test, corrected for the number of tests done.
The grouped data contains the signficance when all lines in a group are treated as 1 sample, compared to the WT.
When either WT or the target line is not measured on a day, the cell is left blank.

Traits are ordered alphabetically

Traits	measuring device
projPlantSurfaceArea_mm2	RGB
convexHullPerimeter_mm	RGB
plantAreaByCircumference	RGB
plantCompactness	RGB
plantConvexHullArea_mm2	RGB
plantPerimeter_mm	RGB
plantStockiness	RGB
plantSurfaceCoverage	RGB
hue	RGB
saturation	RGB
colorvalue	RGB
plantAverageBlue	RGB
plantAverageGreen	RGB
plantAverageRed	RGB
darkyield_stddev	Fluoresence
excentricity	Fluoresence
F0	Fluoresence
Fm	Fluoresence
Fv_Fm	Fluoresence
insideDiameter	Fluoresence
midyieldFluorescenceMeasureme	Fluoresence
minLeafDiameter	Fluoresence
outsideDiameter	Fluoresence

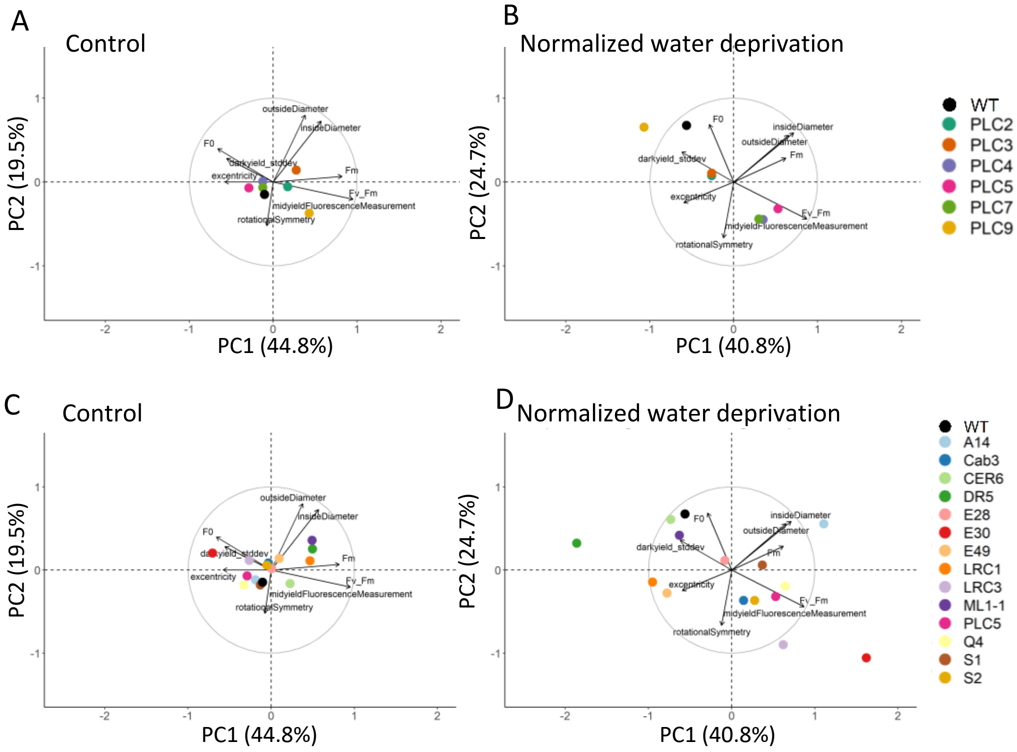

Supplemental Figure 1. PCA plot of Chlorophyl fluorescence camera traits.
PCA plots from control (panel A and C; 21-45 DAS) and water-limiting conditions normalized to control (panel B and D; 30-38 DAS (day 5-13 of stress). Data was normalized per day and per trait, to account for plants being larger at later days. Arrows size and direction indicate how much each trait contributes to each principal component (PC). Percentages of total variance represented by PC1 and PC2 are shown in parentheses. N = 40-60

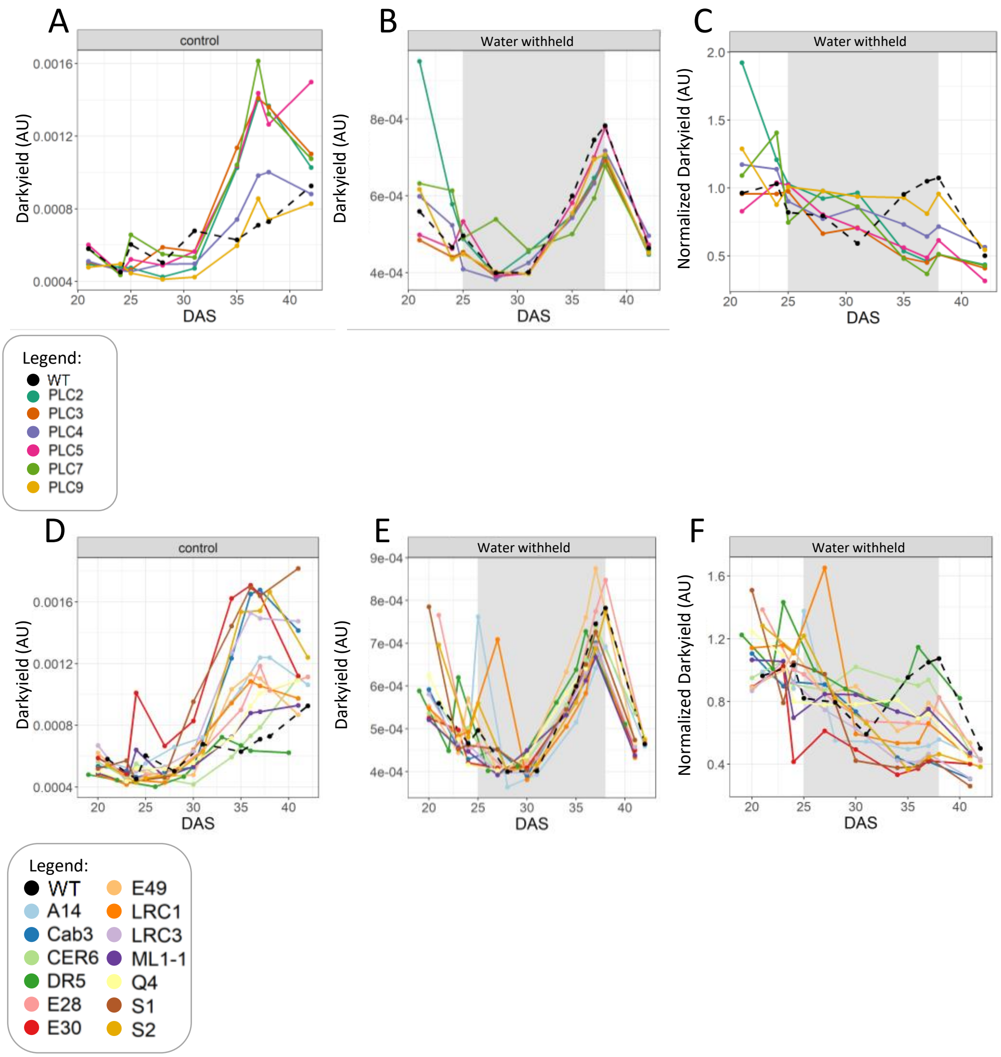

Supplemental Figure 2. Darkyield of plants at control- and water limiting conditions. WT plants were compared to independent transgenic lines in duplo, using 30 replicas each.

A) Darkyield of PLC-OEs at control conditions.

B) Darkyield of PLC-OEs during water withholding, grey shading indicates the period of water withholding from 25 – 38 DAS.

C) Water withholding conditions of PLC-OEs normalized to control conditions.

D) Darkyield of TSEP at control conditions.

E) Darkyield of TSEP during water withholding, grey shading indicates the period of water withholding from 25 – 38 DAS.

F) Water withholding conditions normalized to control conditions.

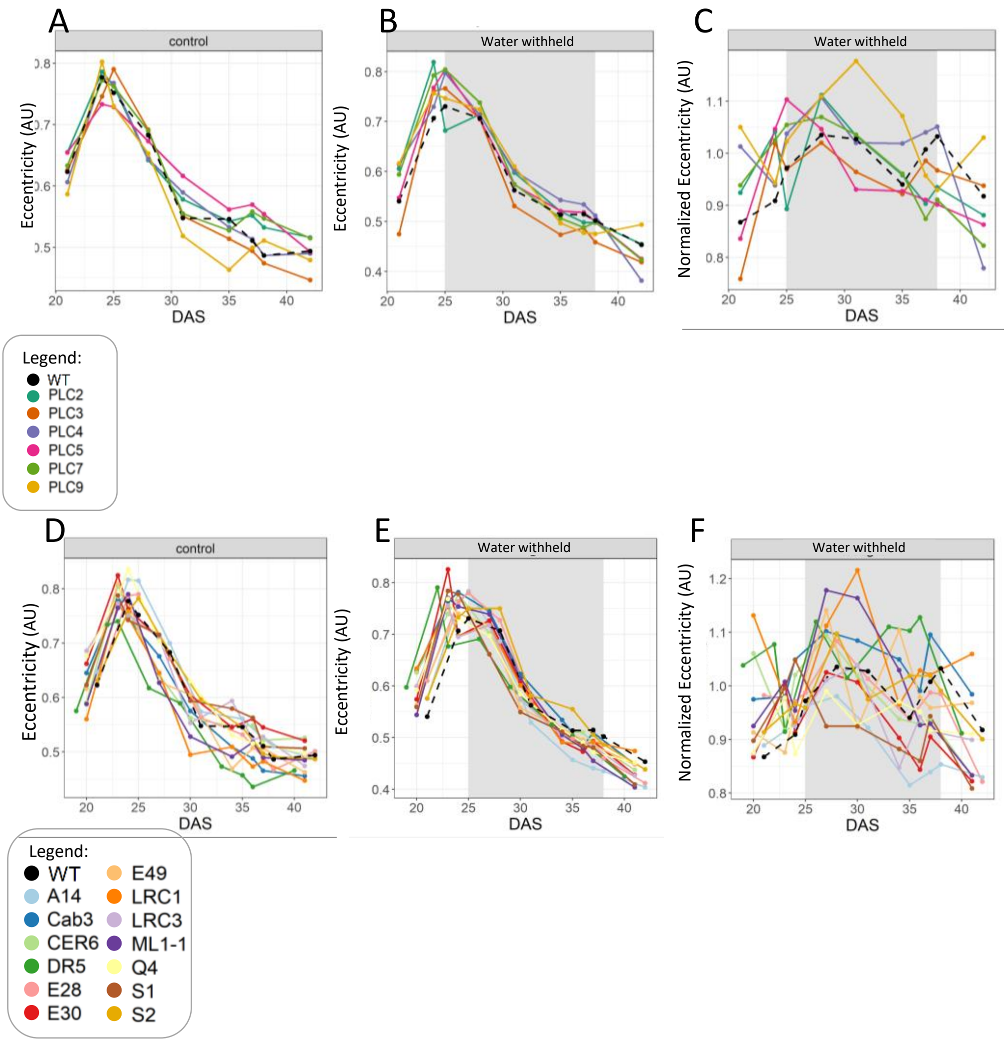

Supplemental Figure 3. Eccentricity of plants at control- and water limiting conditions. WT plants were compared to independent transgenic lines in duplo, using 30 replicas each.

A) Eccentricity pf PLC-OEs at control conditions.

B) Eccentricity of PLC-OEs during water withholding, grey shading indicates the period of water withholding from 25 – 38 DAS.

C) Water withholding conditions of PLC-OEs normalized to control conditions.

D) Eccentricity of TSEP at control conditions.

E) Eccentricity of TSEP during water withholding, grey shading indicates the period of water withholding from 25 – 38 DAS.

F) Water withholding conditions normalized to control conditions.

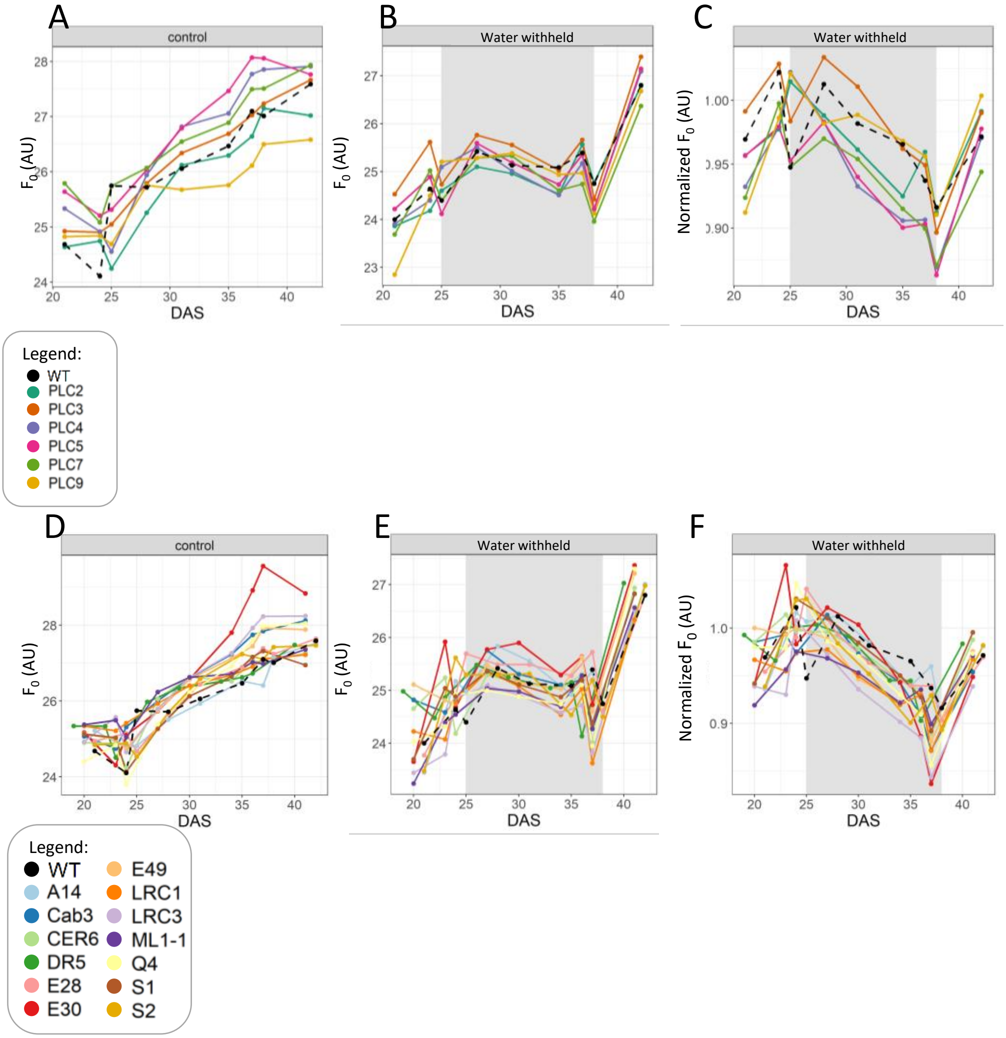

Supplemental Figure 4. F_0 of plants at control- and water limiting conditions.
WT plants were compared to independent transgenic lines in duplo, using 30 replicas each.
A) F_0 pf PLC-OEs at control conditions.
B) F_0 of PLC-OEs during water withholding, grey shading indicates the period of water withholding from 25 – 38 DAS.
C) Water withholding conditions of PLC-OEs normalized to control conditions.
D) F_0 of TSEP at control conditions.
E) F_0 of TSEP during water withholding, grey shading indicates the period of water withholding from 25 – 38 DAS.
F) Water withholding conditions normalized to control conditions.

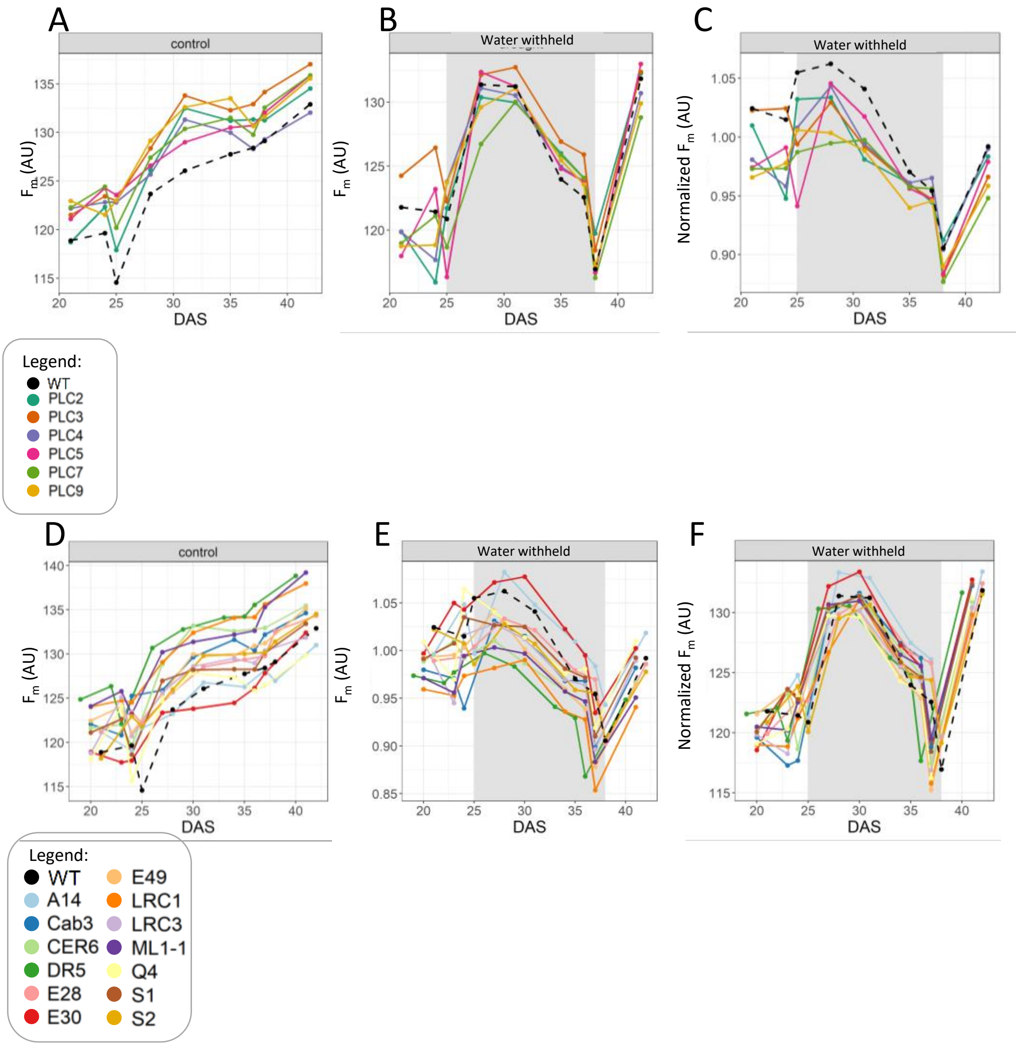

Supplemental Figure 5. F_m of plants at control- and water limiting conditions.
WT plants were compared to independent transgenic lines in duplo, using 30 replicas each.

A) F_m pf PLC-OEs at control conditions.

B) F_m of PLC-OEs during water withholding, grey shading indicates the period of water withholding from 25 – 38 DAS.

C) Water withholding conditions of PLC-OEs normalized to control conditions.

D) F_m of TSEP at control conditions.

E) F_m of TSEP during water withholding, grey shading indicates the period of water withholding from 25 – 38 DAS.

F) Water withholding conditions normalized to control conditions.

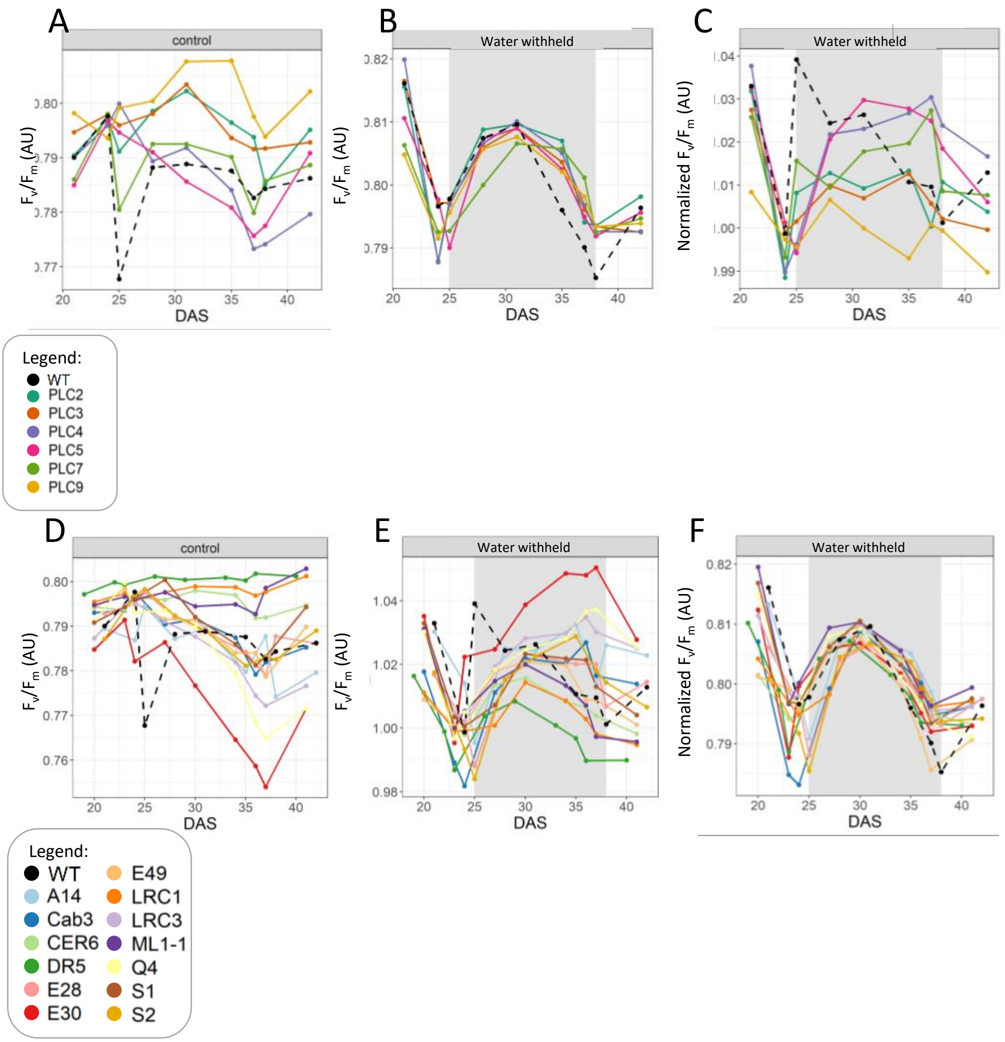

Supplemental Figure 6. F_v/F_m ratios of plants at control- and water limiting conditions. WT plants were compared to independent transgenic lines in duplo, using 30 replicas each.

A) F_v/F_m ratios of PLC-OEs at control conditions.

B) F_v/F_m ratios of PLC-OEs during water withholding, grey shading indicates the period of water withholding from 25 – 38 DAS.

C) Water withholding conditions of PLC-OEs normalized to control conditions.

D) F_v/F_m ratios of TSEP at control conditions.

E) F_v/F_m ratios of TSEP during water withholding, grey shading indicates the period of water withholding from 25 – 38 DAS.

F) Water withholding conditions normalized to control conditions.

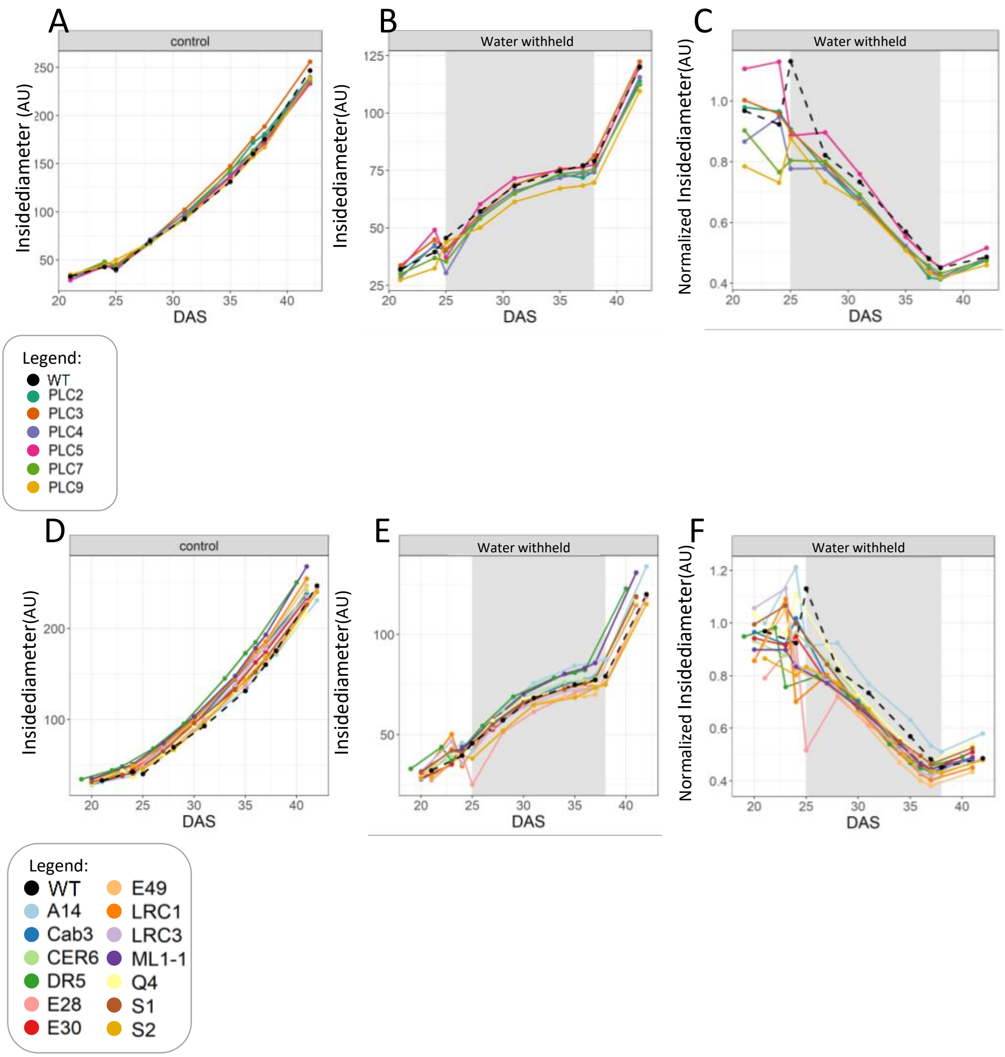

Supplemental Figure 7. Insidediameter of plants at control- and water limiting conditions.

WT plants were compared to independent transgenic lines in duplo, using 30 replicas each.

A) Insidediameter of PLC-OEs at control conditions.

B) Insidediameter of PLC-OEs during water withholding, grey shading indicates the period of water withholding from 25 – 38 DAS.

C) Water withholding conditions of PLC-OEs normalized to control conditions.

D) Insidediameter of TSEP at control conditions.

E) Insidediameter of TSEP during water withholding, grey shading indicates the period of water withholding from 25 – 38 DAS.

F) Water withholding conditions normalized to control conditions.

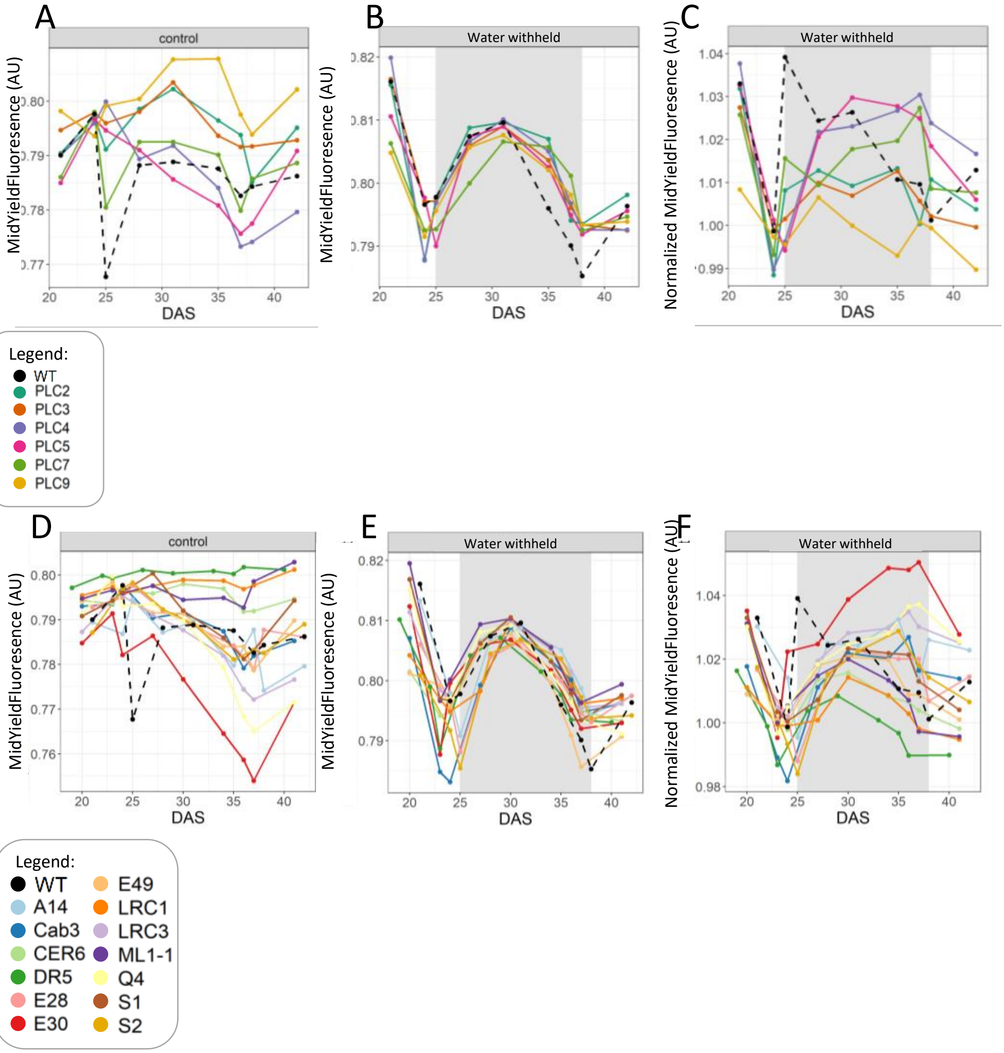

Supplemental Figure 8. midyieldfluoresence of plants at control- and water limiting conditions.

WT plants were compared to independent transgenic lines in duplo, using 30 replicas each.

A) midyieldfluoresence of PLC-OEs at control conditions.

B) midyieldfluoresence of PLC-OEs during water withholding, grey shading indicates the period of water withholding from 25 – 38 DAS.

C) Water withholding conditions of PLC-OEs normalized to control conditions.

D) midyieldfluoresence of TSEP at control conditions.

E) midyieldfluoresence of TSEP during water withholding, grey shading indicates the period of water withholding from 25 – 38 DAS.

F) Water withholding conditions normalized to control conditions.

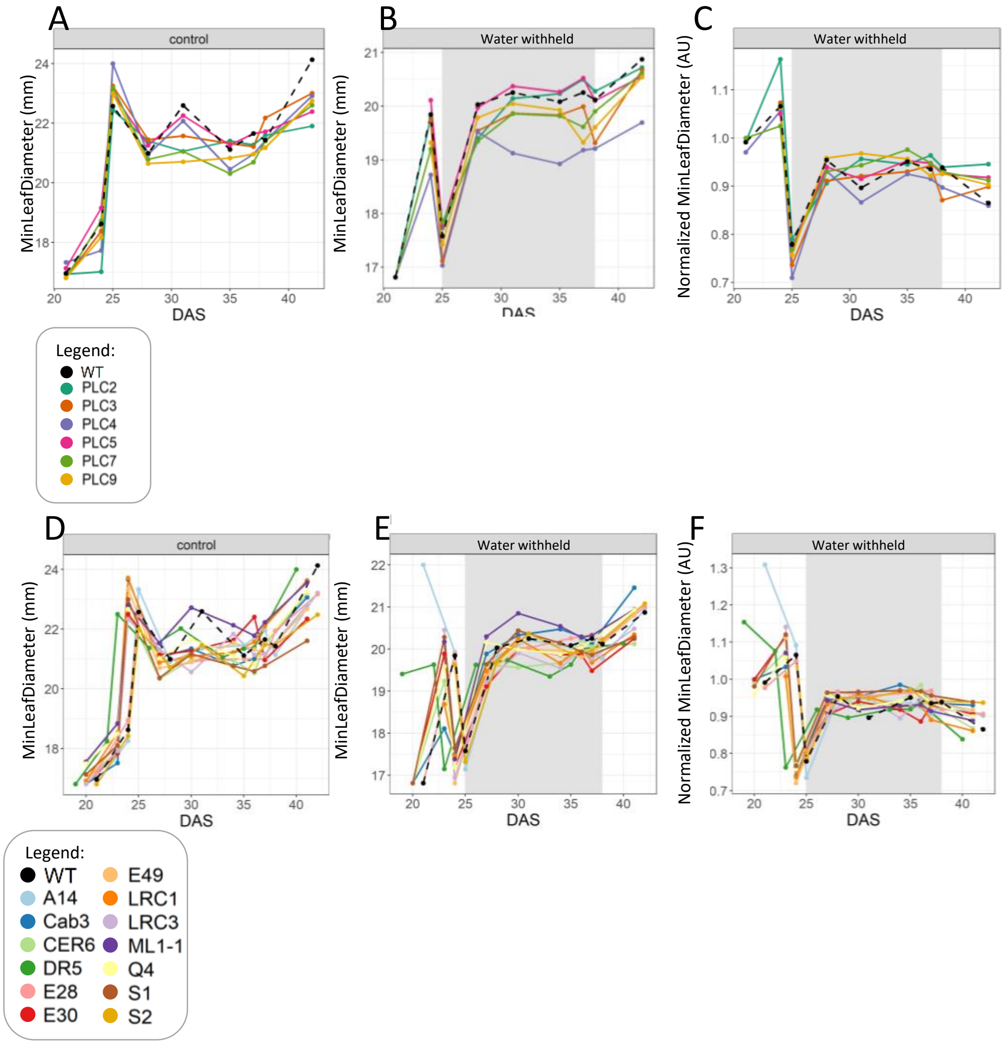

Supplemental Figure 9. Minimal leaf diameter of plants at control- and water limiting conditions.

WT plants were compared to independent transgenic lines in duplo, using 30 replicas each.

A) Minimal leaf diameter of PLC-OEs at control conditions.

B) Minimal leaf diameter of PLC-OEs during water withholding, grey shading indicates the period of water withholding from 25 – 38 DAS.

C) Water withholding conditions of PLC-OEs normalized to control conditions.

D) Minimal leaf diameter of TSEP at control conditions.

E) Minimal leaf diameter of TSEP during water withholding, grey shading indicates the period of water withholding from 25 – 38 DAS.

F) Water withholding conditions normalized to control conditions.

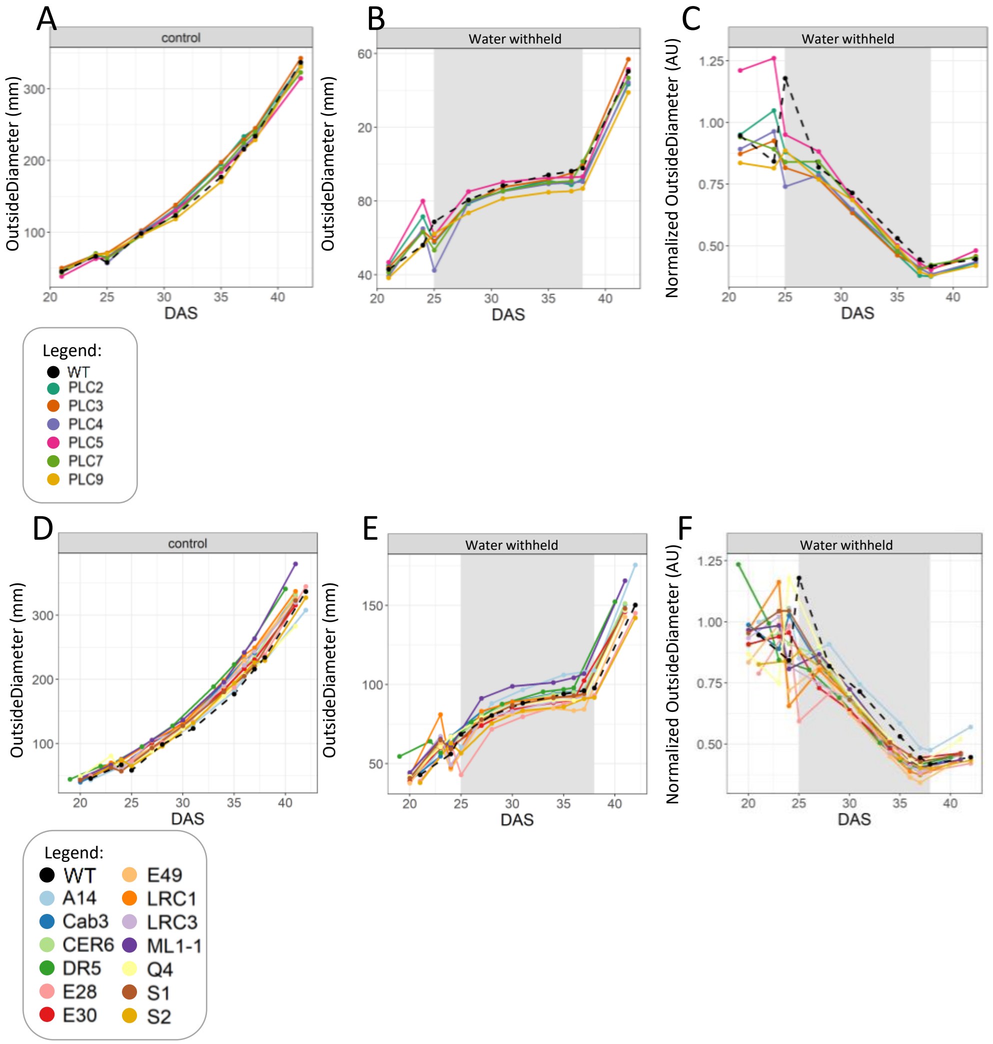

Supplemental Figure 10. Outside leaf diameter of plants at control- and water limiting conditions.

WT plants were compared to independent transgenic lines in duplo, using 30 replicas each.

A) Outside leaf diameter of PLC-OEs at control conditions.

B) Outside leaf diameter of PLC-OEs during water withholding, grey shading indicates the period of water withholding from 25 – 38 DAS.

C) Water withholding conditions of PLC-OEs normalized to control conditions.

D) Outside leaf diameter of TSEP at control conditions.

E) Outside leaf diameter of TSEP during water withholding, grey shading indicates the period of water withholding from 25 – 38 DAS.

F) Water withholding conditions normalized to control conditions.

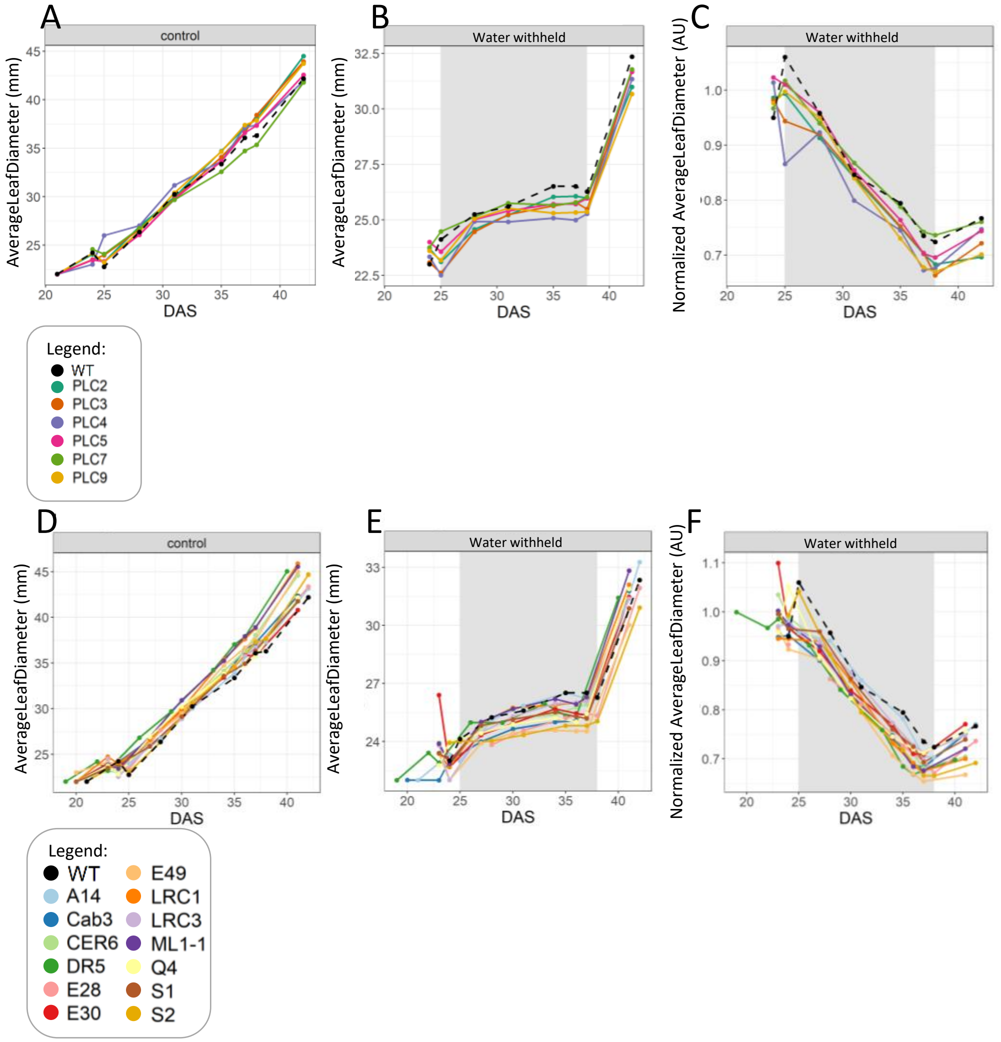

Supplemental Figure 11. Average leaf diameter of plants at control- and water limiting conditions.

WT plants were compared to independent transgenic lines in duplo, using 30 replicas each.

A) Average leaf diameter of PLC-OEs at control conditions.

B) Average leaf diameter of PLC-OEs during water withholding, grey shading indicates the period of water withholding from 25 – 38 DAS.

C) Water withholding conditions of PLC-OEs normalized to control conditions.

D) Average leaf diameter of TSEP at control conditions.

E) Average leaf diameter of TSEP during water withholding, grey shading indicates the period of water withholding from 25 – 38 DAS.

F) Water withholding conditions normalized to control conditions.

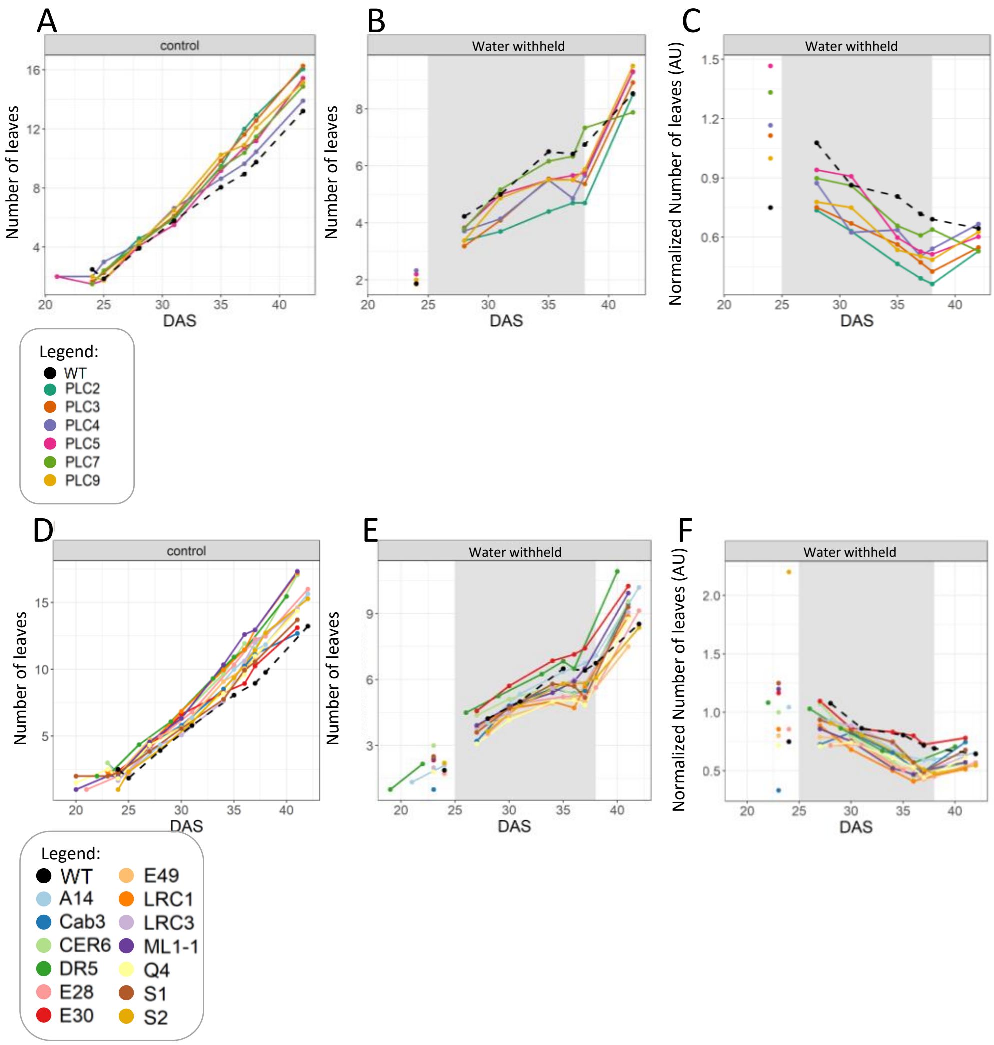

Supplemental Figure 12. Number of leaves of plants at control- and water limiting conditions.

WT plants were compared to independent transgenic lines in duplo, using 30 replicas each.

A) Number of leaves of PLC-OEs at control conditions.

B) Number of leaves of PLC-OEs during water withholding, grey shading indicates the period of water withholding from 25 – 38 DAS.

C) Water withholding conditions of PLC-OEs normalized to control conditions.

D) Number of leaves of TSEP at control conditions.

E) Number of leaves of TSEP during water withholding, grey shading indicates the period of water withholding from 25 – 38 DAS.

F) Water withholding conditions normalized to control conditions.

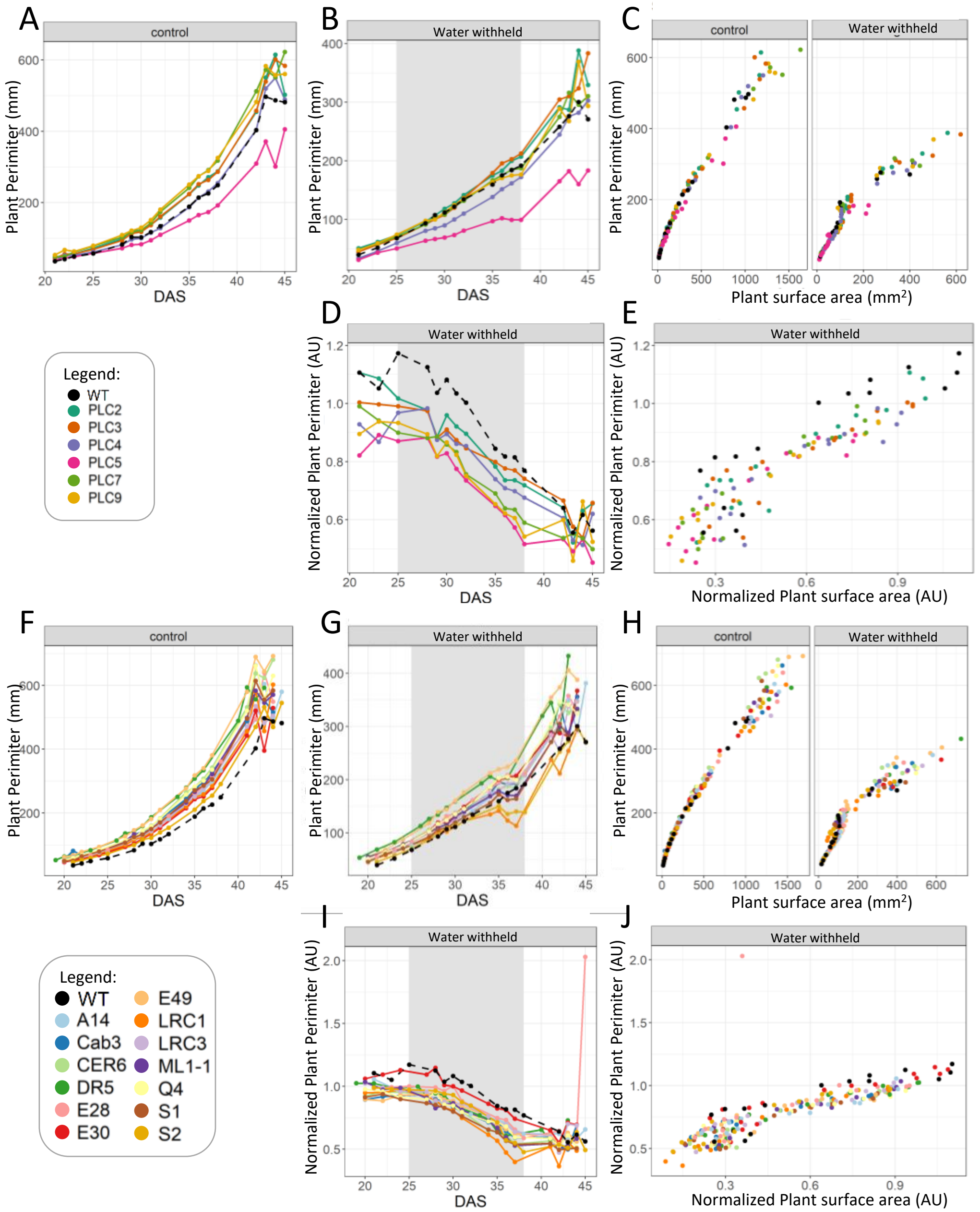

Supplemental figure 13. Plant perimeter at control- and water limiting conditions.
The Plant perimeter was measured for:
A, F) PLC-OE and TSEP grown at control conditions.
B, G) PLC-OE and TSEP at water-withholding conditions, with grey shading indicating period of water withholding (25-38 DAS).
C, H) PLC-OE and TSEP in Water-withholding conditions normalized to control per day
D, I) PLC-OE and TSEP in Water withholding conditions normalized to control conditions.
E, J) PLC-OE and TSEP in water withholding conditions normalized to Projected plant surface area

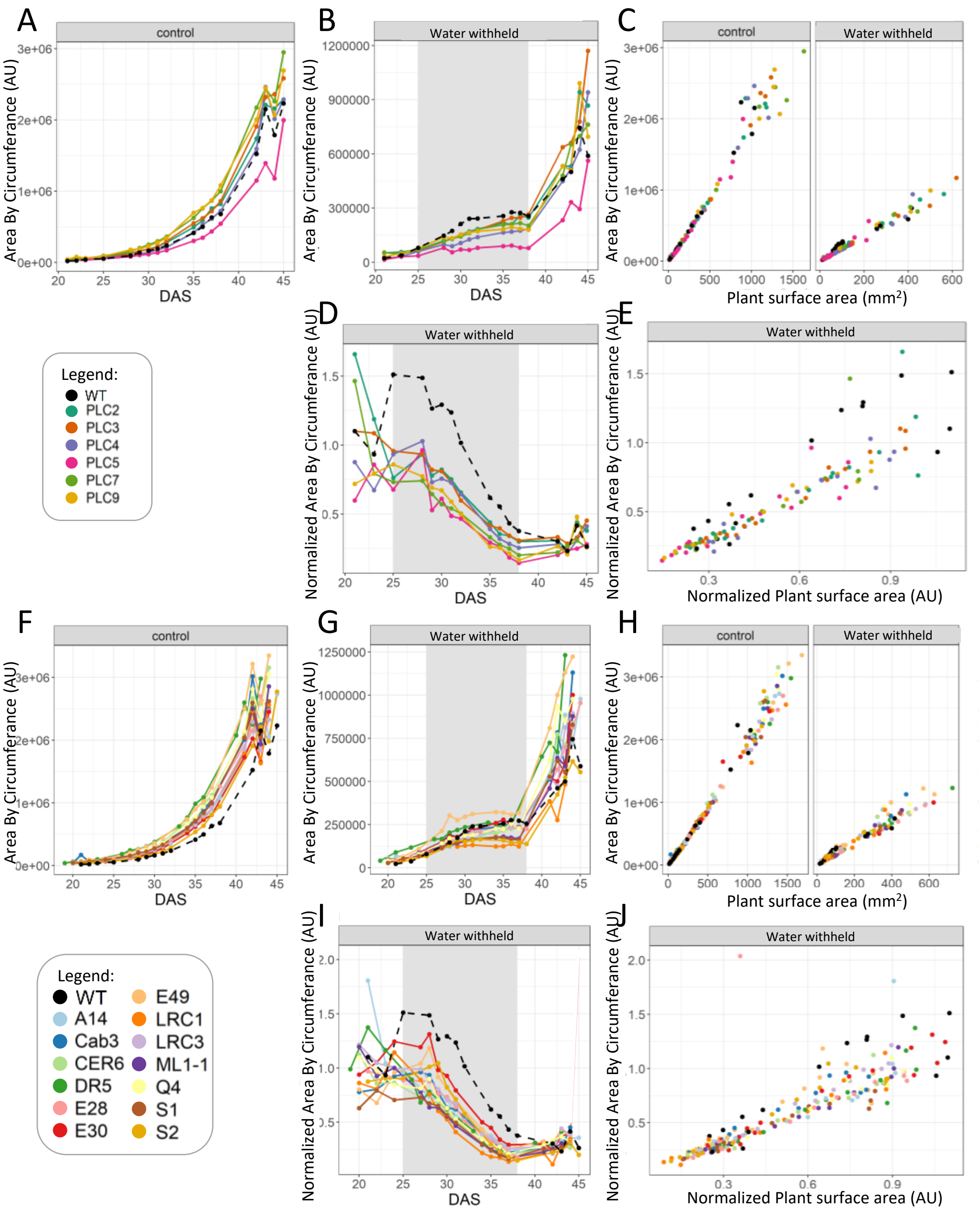

Supplemental figure 14. Area by circumference at control- and water limiting conditions. The Area by circumference was measured for:

A, F) PLC-OE and TSEP grown at control conditions.

B, G) PLC-OE and TSEP at water-withholding conditions, with grey shading indicating period of water withholding (25-38 DAS).

C, H) PLC-OE and TSEP in Water-withholding conditions normalized to control per day

D, I) PLC-OE and TSEP in Water withholding conditions normalized to control conditions.

E, J) PLC-OE and TSEP in water withholding conditions normalized to Projected plant surface area

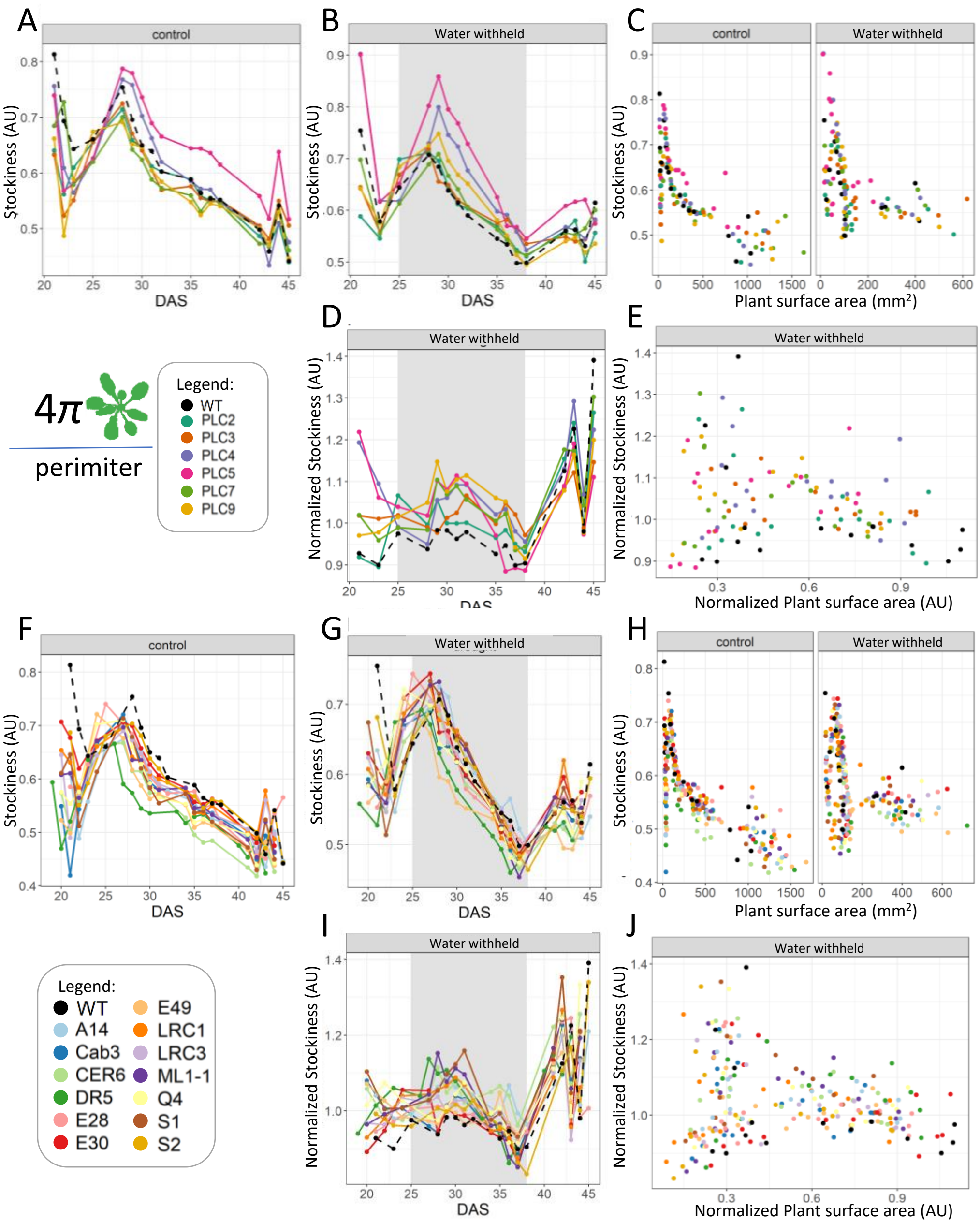

Supplemental figure 15. Stockiness at control- and water limiting conditions.
The stockiness was measured for:
A, F) PLC-OE and TSEP grown at control conditions.
B, G) PLC-OE and TSEP at water-withholding conditions, with grey shading indicating period of water withholding (25-38 DAS).
C, H) PLC-OE and TSEP in Water-withholding conditions normalized to control per day
D, I) PLC-OE and TSEP in Water withholding conditions normalized to control conditions.
E, J) PLC-OE and TSEP in water withholding conditions normalized to Projected plant surface area

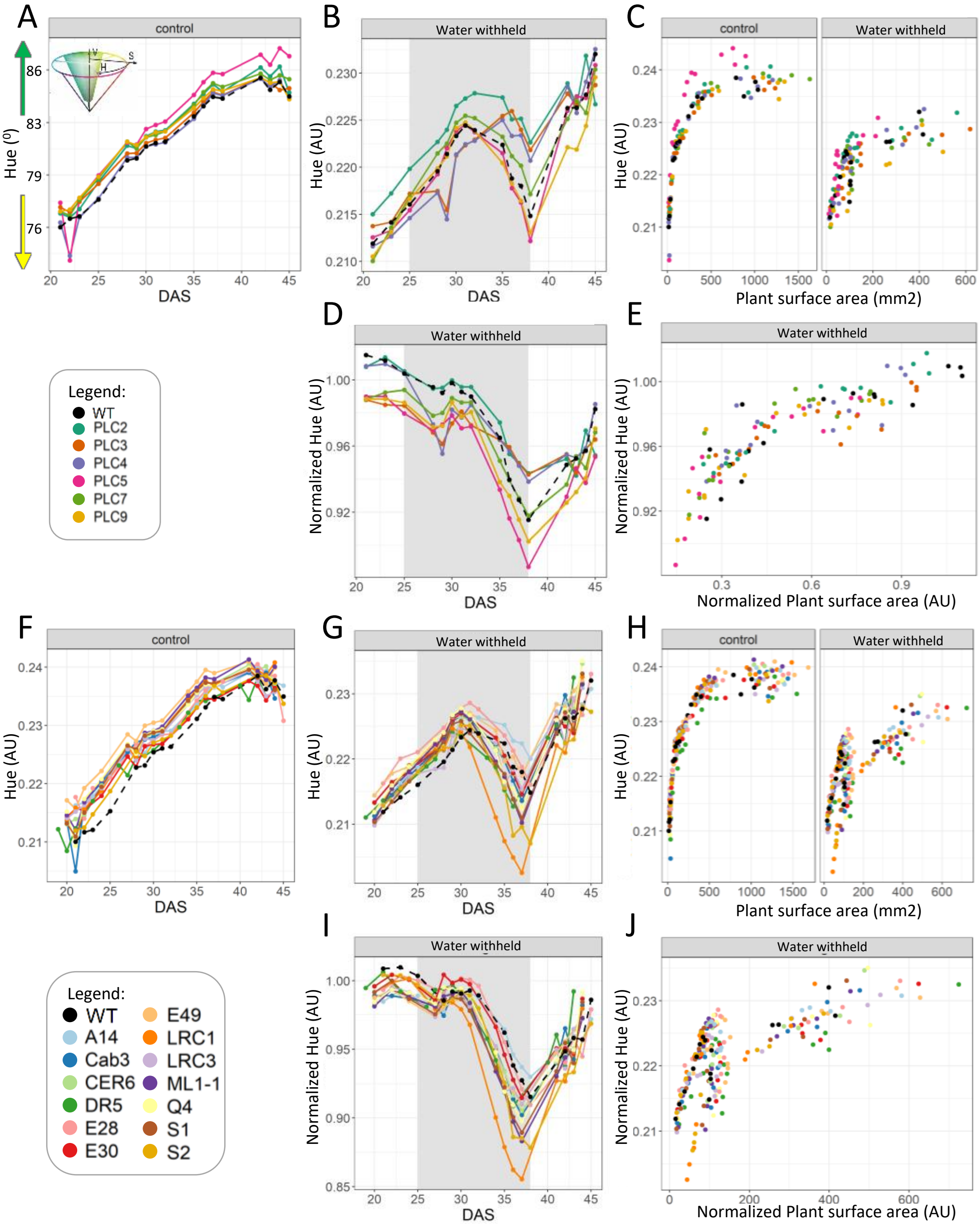

Supplemental figure 16. Hue at control- and water limiting conditions.
The Hue was measured for :
A, F) PLC-OE and TSEP grown at control conditions.
B, G) PLC-OE and TSEP at water-withholding conditions, with grey shading indicating period of water withholding (25-38 DAS).
C, H) PLC-OE and TSEP in Water-withholding conditions normalized to control per day
D, I) PLC-OE and TSEP in Water withholding conditions normalized to control conditions.
E, J) PLC-OE and TSEP in water withholding conditions normalized to Projected plant surface area

Brightness PLC

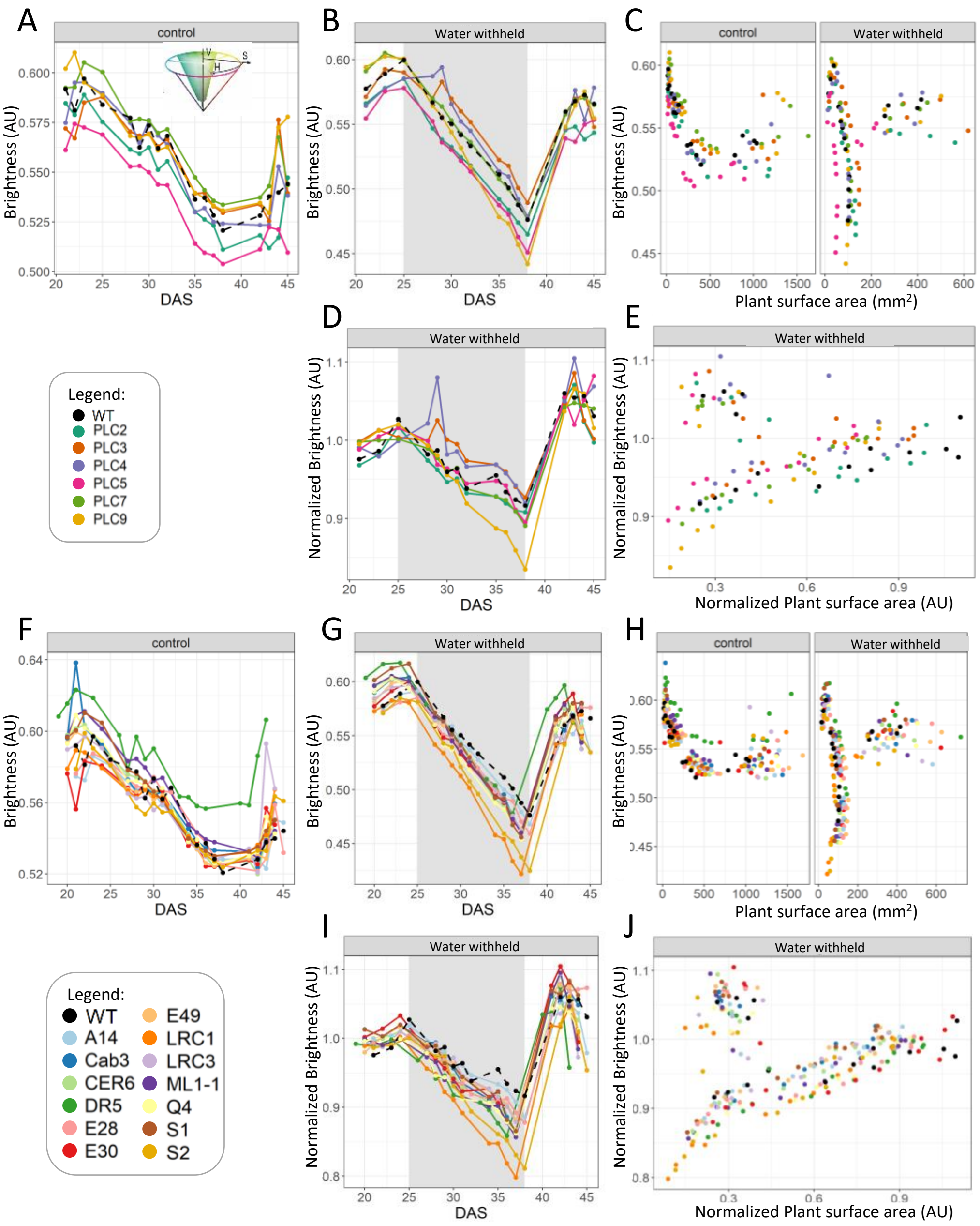

Supplemental figure 17. brightness at control- and water limiting conditions.
The brightness was measured for :
A, F) PLC-OE and TSEP grown at control conditions.
B, G) PLC-OE and TSEP at water-withholding conditions, with grey shading indicating period of water withholding (25-38 DAS).
C, H) PLC-OE and TSEP in Water-withholding conditions normalized to control per day
D, I) PLC-OE and TSEP in Water withholding conditions normalized to control conditions.
E, J) PLC-OE and TSEP in water withholding conditions normalized to Projected plant surface area

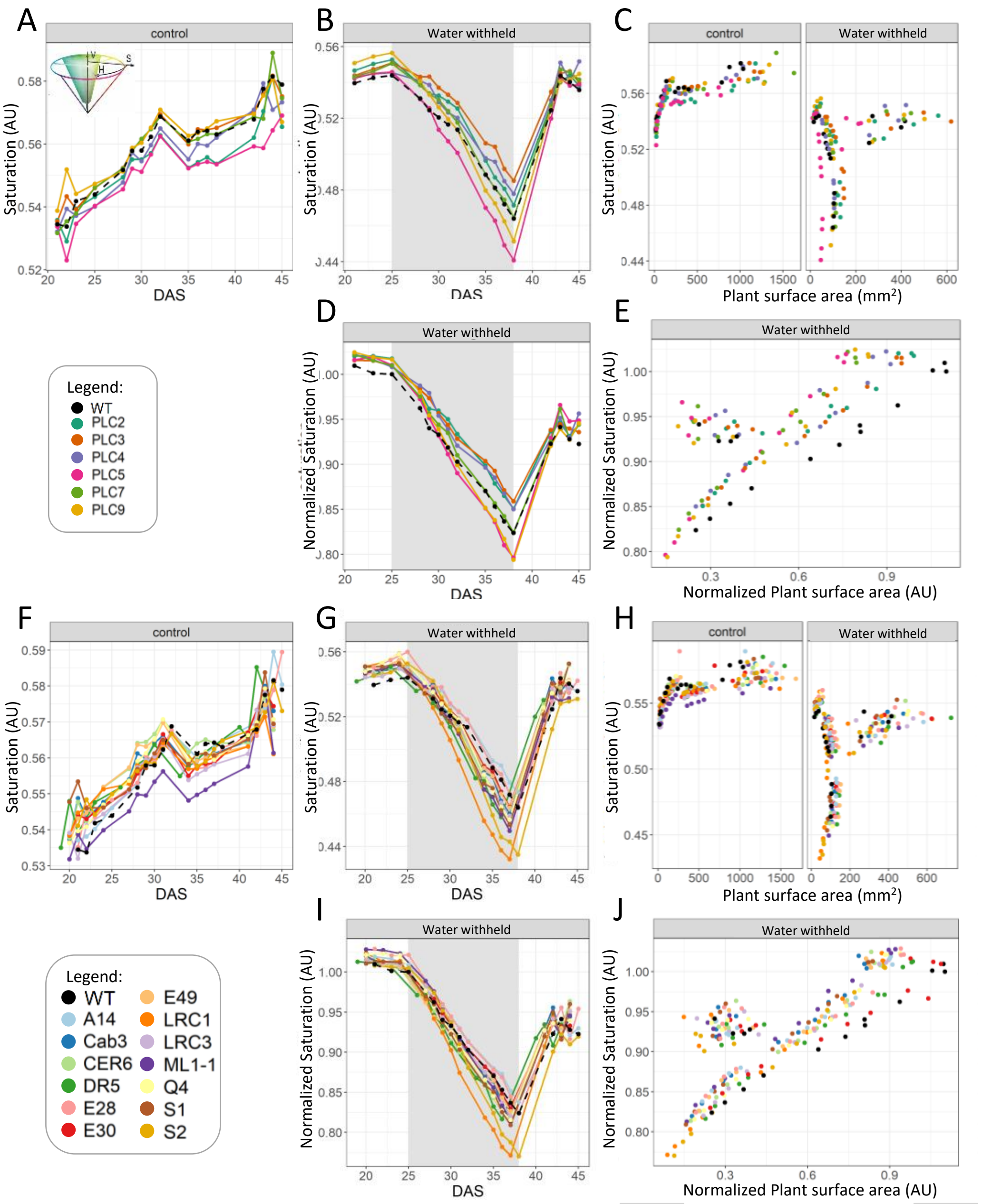

Supplemental figure 18. Saturation at control- and water limiting conditions.
The Saturation was measured for :
A, F) PLC-OE and TSEP grown at control conditions.
B, G) PLC-OE and TSEP at water-withholding conditions, with grey shading indicating period of water withholding (25-38 DAS).
C, H) PLC-OE and TSEP in Water-withholding conditions normalized to control per day
D, I) PLC-OE and TSEP in Water withholding conditions normalized to control conditions.
E, J) PLC-OE and TSEP in water withholding conditions normalized to Projected plant surface area

Figure 17

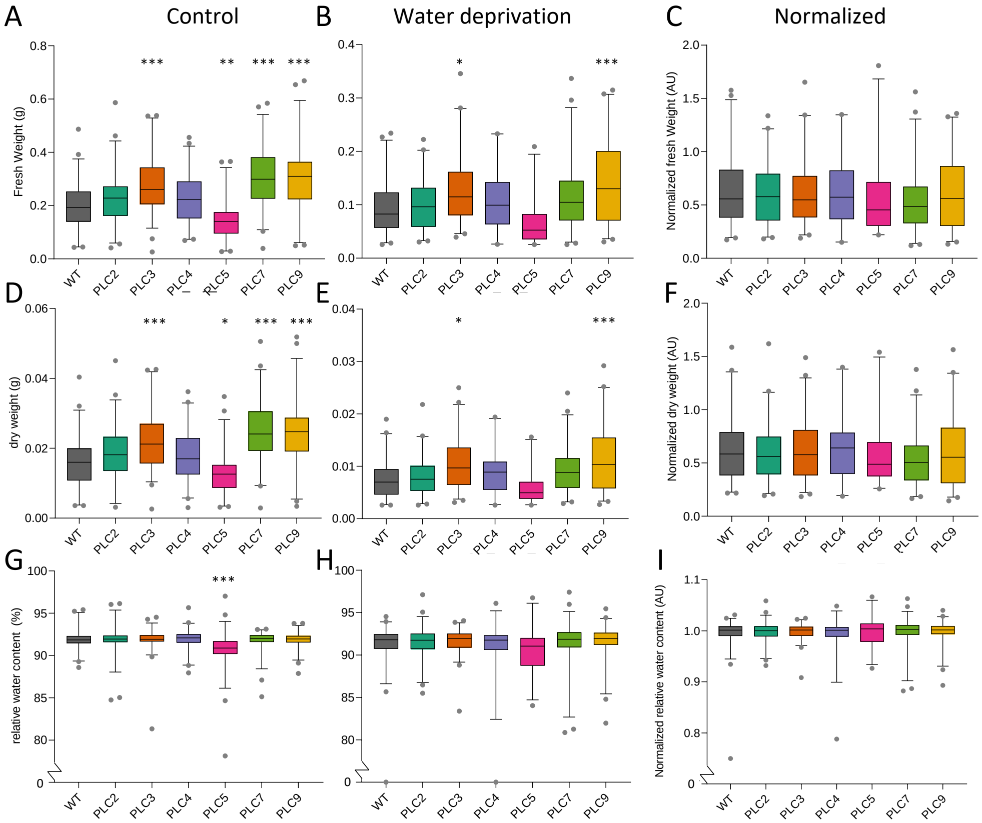

Supplemental figure 19. Fresh- and dry weight and relative water content in WT and PLC-OE lines.
Plants were harvested and measured at 45 DAS. Water-stressed plants were deprived of water for 13 days, and left to recover for seven days before harvest. After measuring fresh weight (FW), plants were dried for 48 hours in a 70° oven and measured for their dry weight (DW). RWC was determined by the formula: (FW-DW)/FW*100%.
A-C) Fresh weights (control-, drought-, or drought normalized to control conditions, respectively).
D-F) Dry weights
G-I) RWC (A-F)
Significance was determined by one-way ANOVA, with multiple comparison test Dunnet. *P < 0.05, P**<0.01,***P < 0.001

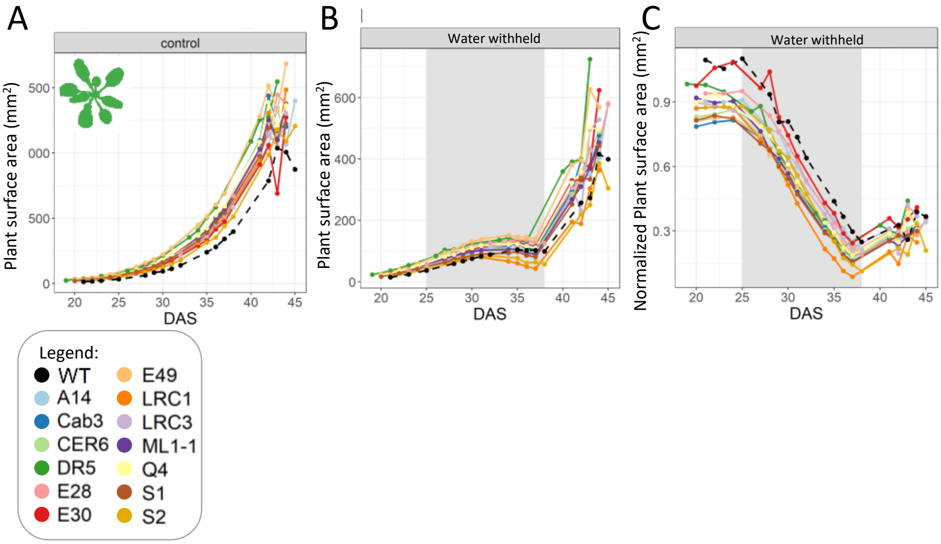

Supplemental figure 20. Projected plant surface area of TSEP lines under control and water limiting conditions.

Arabidopsis WT and TSEP lines plants were monitored using the GROWSCREEN-FLUORO system for indicated times. For each genotype, two different insertion lines with 30 replicates were used.

A) Projected leaf area (schematically drawn in upper left corner) at control conditions.

B) Projected leaf area at water-withholding conditions (25-37 DAS).

C) Projected leaf area at water-withholding conditions normalized to control.

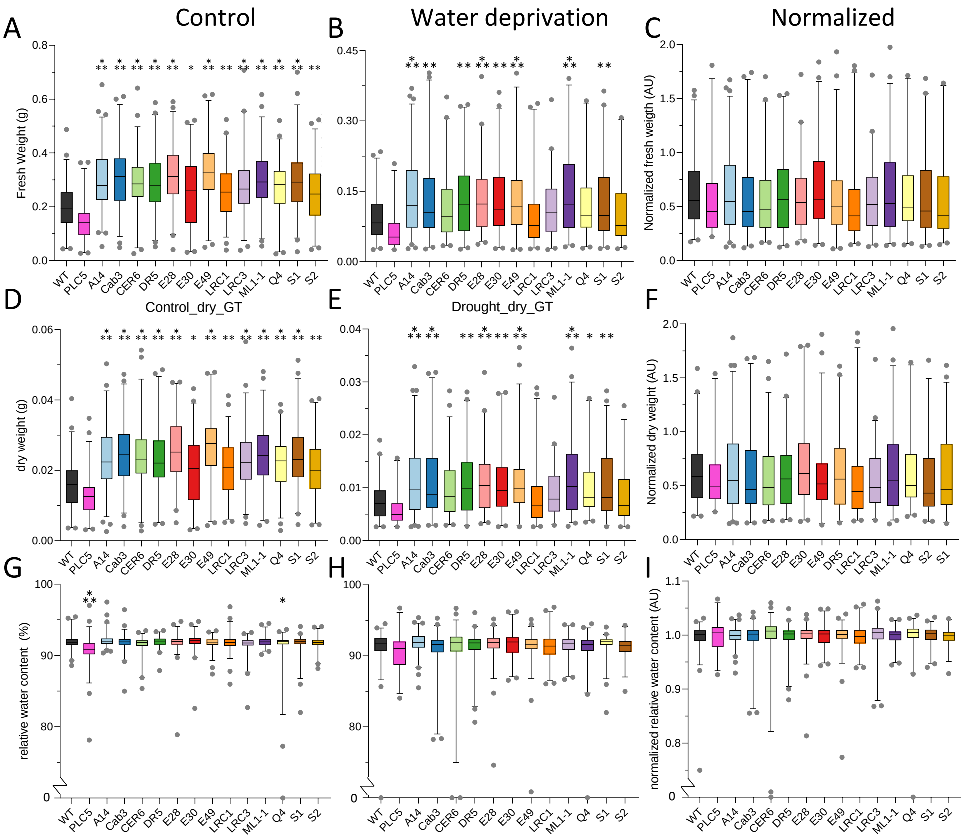

Supplemental figure 21. Fresh- and dry weight and relative water content in WT and TSEP lines.

Plants were harvested and measured at 45 DAS. Water-stressed plants were deprived of water for 13 days, and left to recover for seven days before harvest. After measuring fresh weight (FW), plants were dried for 48 hours in a 70° oven and measured for their dry weight (DW). RWC was determined by the formula: (FW-DW)/FW*100%.

A-C) Fresh weights (control-, drought-, or drought normalized to control conditions, respectively).

D-F) Dry weights

G-I) RWC (A-F)

Significance was determined by one-way ANOVA, with multiple comparison test Dunnet. *P < 0.05, P**<0.01,***P < 0.001

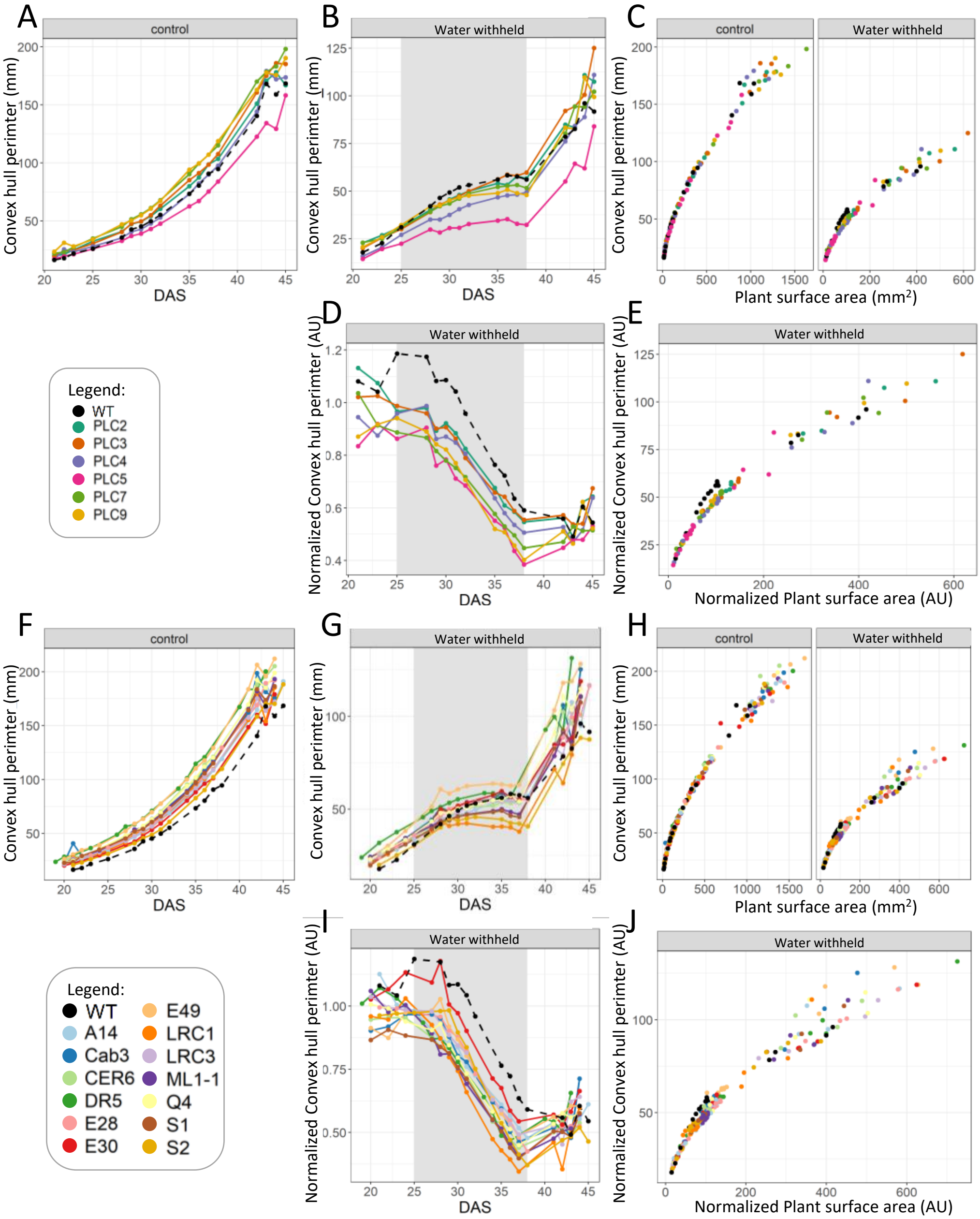

Supplemental figure 22. Convex hull permiter at control- and water limiting conditions.
The Convex hull permiter was measured for:
A, F) PLC-OE and TSEP grown at control conditions.
B, G) PLC-OE and TSEP at water-withholding conditions, with grey shading indicating period of water withholding (25-38 DAS).
C, H) PLC-OE and TSEP in Water-withholding conditions normalized to control per day
D, I) PLC-OE and TSEP in Water withholding conditions normalized to control conditions.
E, J) PLC-OE and TSEP in water withholding conditions normalized to Projected plant surface area

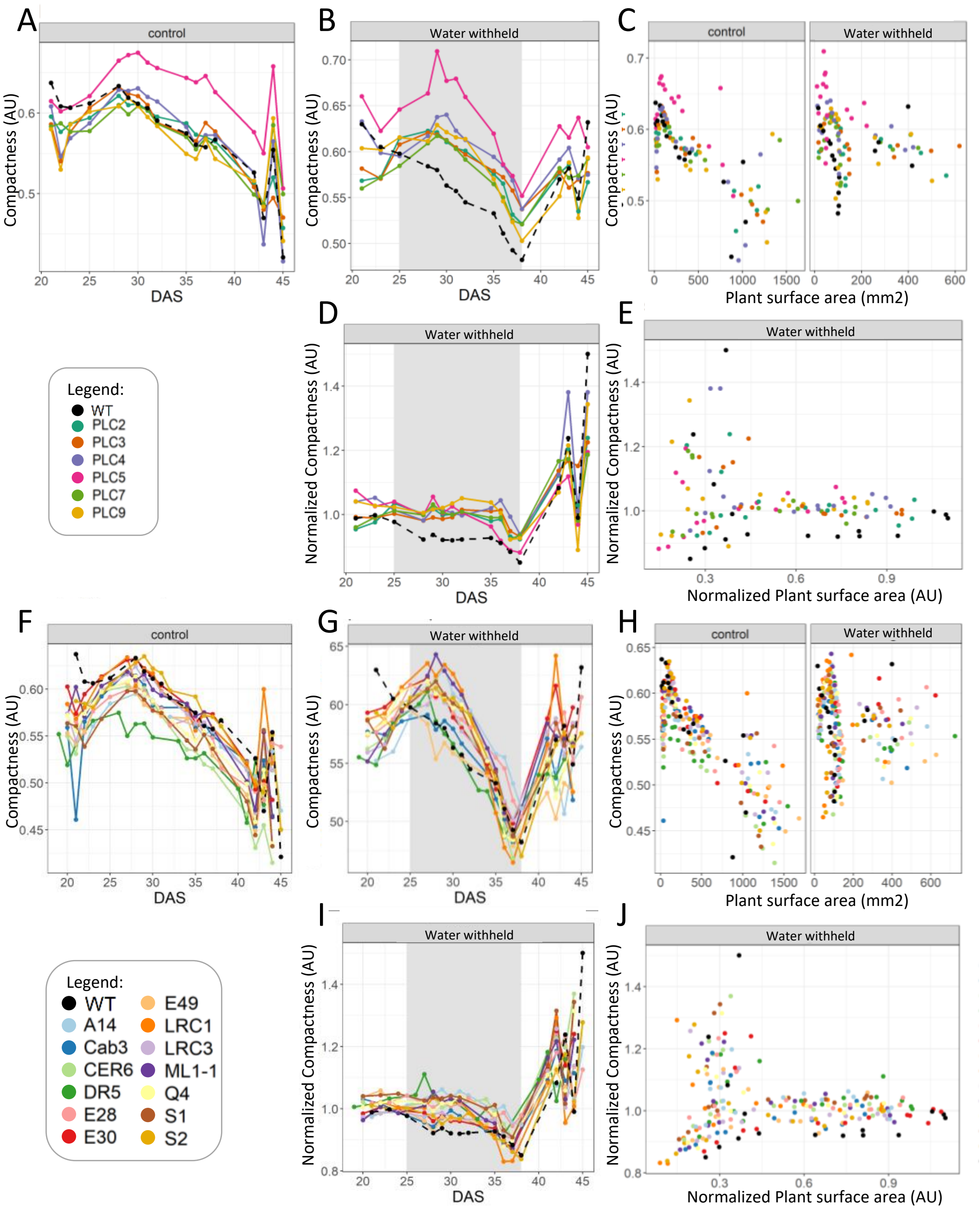

Supplemental figure 23. compactness at control- and water limiting conditions.
The compactness was measured for:
A, F) PLC-OE and TSEP grown at control conditions.
B, G) PLC-OE and TSEP at water-withholding conditions, with grey shading indicating period of water withholding (25-38 DAS).
C, H) PLC-OE and TSEP in Water-withholding conditions normalized to control per day
D, I) PLC-OE and TSEP in Water withholding conditions normalized to control conditions.
E, J) PLC-OE and TSEP in water withholding conditions normalized to Projected plant surface area

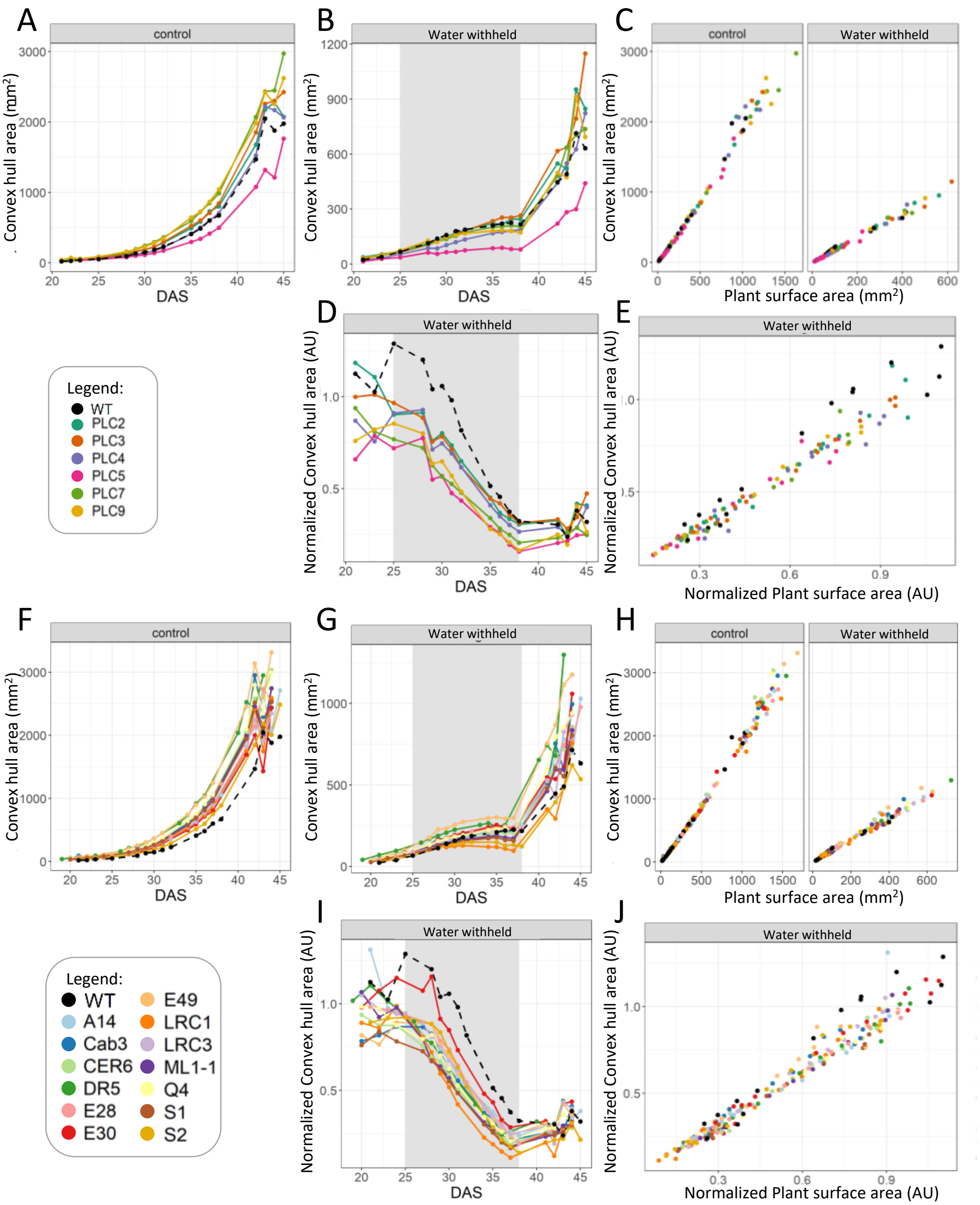

Supplemental figure 24. Convex hull area at control- and water limiting conditions.
The Convex hull area was measured for:
A, F) PLC-OE and TSEP grown at control conditions.
B, G) PLC-OE and TSEP at water-withholding conditions, with grey shading indicating period of water withholding (25-38 DAS).
C, H) PLC-OE and TSEP in Water-withholding conditions normalized to control per day
D, I) PLC-OE and TSEP in Water withholding conditions normalized to control conditions.
E, J) PLC-OE and TSEP in water withholding conditions normalized to Projected plant surface area

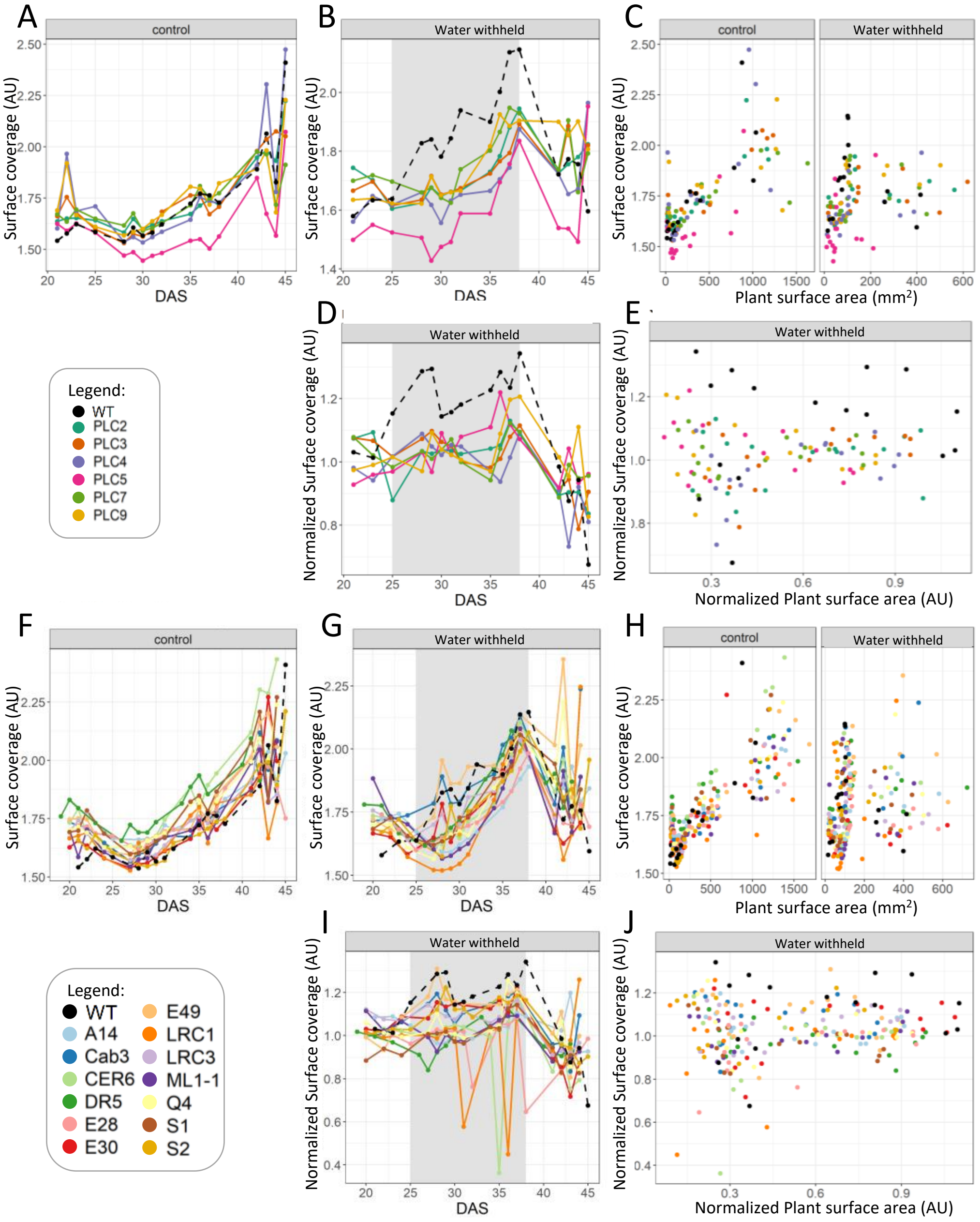

Supplemental figure 25. Surface coverage at control- and water limiting conditions.
The Surface coverage was measured for:
A, F) PLC-OE and TSEP grown at control conditions.
B, G) PLC-OE and TSEP at water-withholding conditions, with grey shading indicating period of water withholding (25-38 DAS).
C, H) PLC-OE and TSEP in Water-withholding conditions normalized to control per day
D, I) PLC-OE and TSEP in Water withholding conditions normalized to control conditions.
E, J) PLC-OE and TSEP in water withholding conditions normalized to Projected plant surface area

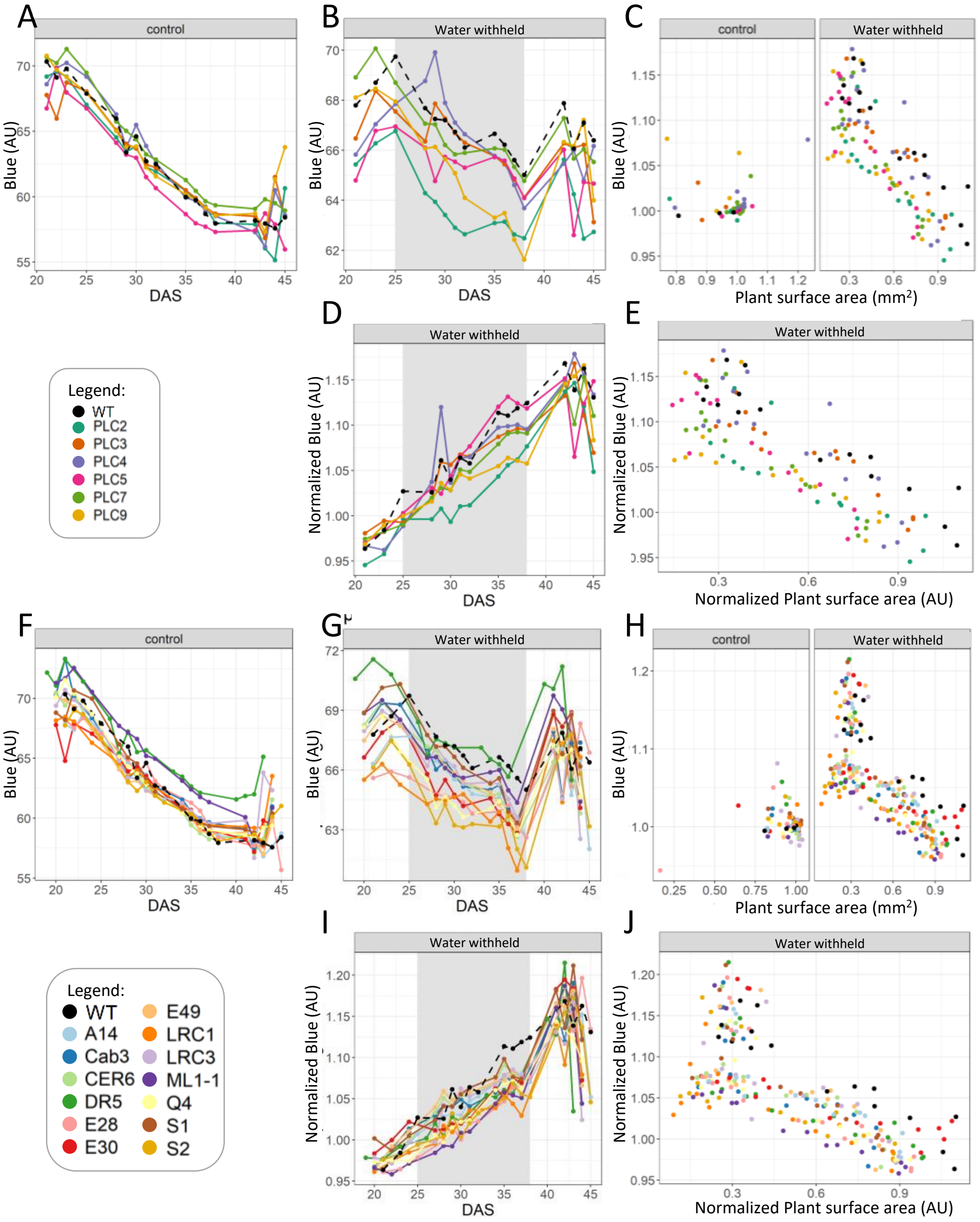

Supplemental figure 26. RGB Blue values at control- and water limiting conditions.
The RGB Blue values was measured for:
A, F) PLC-OE and TSEP grown at control conditions.
B, G) PLC-OE and TSEP at water-withholding conditions, with grey shading indicating period of water withholding (25-38 DAS).
C, H) PLC-OE and TSEP in Water-withholding conditions normalized to control per day
D, I) PLC-OE and TSEP in Water withholding conditions normalized to control conditions.
E, J) PLC-OE and TSEP in water withholding conditions normalized to Projected plant surface area

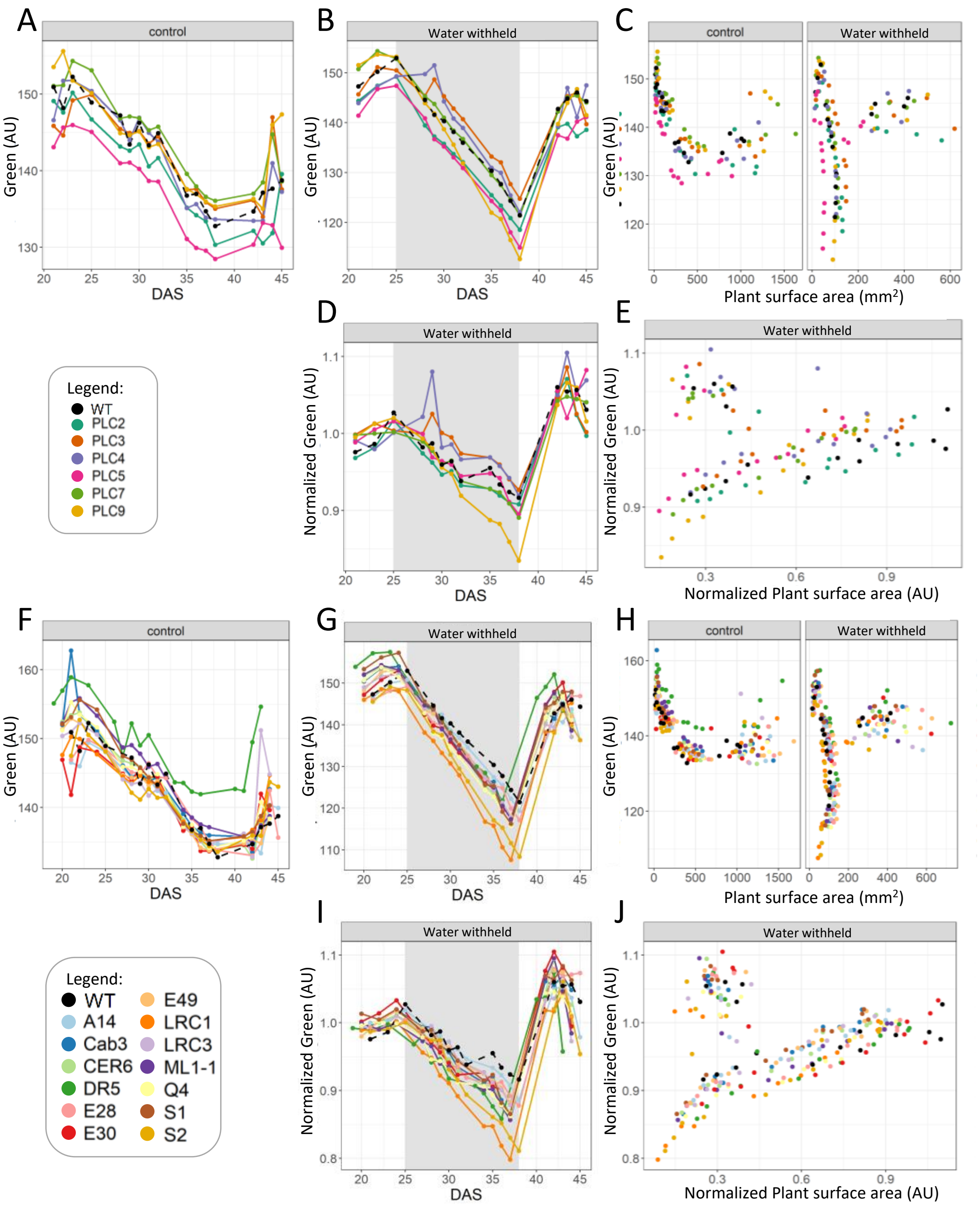

Supplemental figure 27. RGB Green values at control- and water limiting conditions.
The RGB Green values was measured for:
A, F) PLC-OE and TSEP grown at control conditions.
B, G) PLC-OE and TSEP at water-withholding conditions, with grey shading indicating period of water withholding (25-38 DAS).
C, H) PLC-OE and TSEP in Water-withholding conditions normalized to control per day
D, I) PLC-OE and TSEP in Water withholding conditions normalized to control conditions.
E, J) PLC-OE and TSEP in water withholding conditions normalized to Projected plant surface area

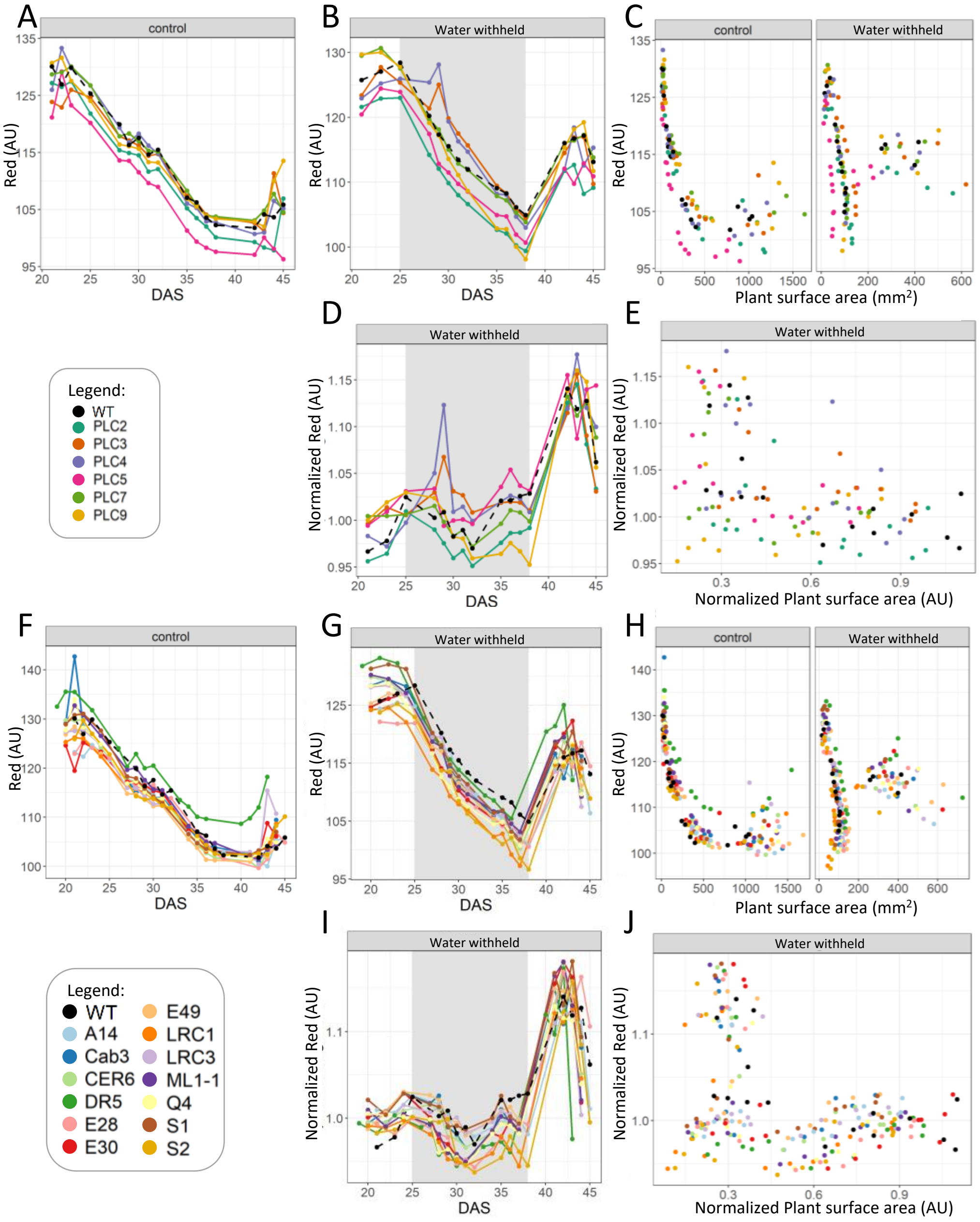

Supplemental figure 28. RGB Red values at control- and water limiting conditions.
The RGB Red values was measured for:
A, F) PLC-OE and TSEP grown at control conditions.
B, G) PLC-OE and TSEP at water-withholding conditions, with grey shading indicating period of water withholding (25-38 DAS).
C, H) PLC-OE and TSEP in Water-withholding conditions normalized to control per day
D, I) PLC-OE and TSEP in Water withholding conditions normalized to control conditions.
E, J) PLC-OE and TSEP in water withholding conditions normalized to Projected plant surface area
